# On the genetic architecture of rapidly adapting and convergent life history traits in guppies

**DOI:** 10.1038/s41437-022-00512-6

**Published:** 2022-03-08

**Authors:** James R. Whiting, Josephine R. Paris, Paul J. Parsons, Sophie Matthews, Yuridia Reynoso, Kimberly A. Hughes, David Reznick, Bonnie A. Fraser

**Affiliations:** 1 Department of Biosciences, University of Exeter, Stocker Road, Exeter, EX4 4QD UK; 2 Department of Biological Sciences, University of Calgary, Calgary, AB T2N 1N4 Canada; 3 Department of Animal and Plant Sciences, University of Sheffield, Alfred Denny Building, Western Bank, Sheffield, S10 2TN UK; 4 Department of Biology, University of California Riverside, Riverside, CA 92521 USA; 5 Department of Biological Science, Florida State University, Tallahassee, FL 32303 USA

**Keywords:** Evolutionary genetics, Quantitative trait

## Abstract

The genetic basis of traits shapes and constrains how adaptation proceeds in nature; rapid adaptation can proceed using stores of polygenic standing genetic variation or hard selective sweeps, and increasing polygenicity fuels genetic redundancy, reducing gene re-use (genetic convergence). Guppy life history traits evolve rapidly and convergently among natural high- and low-predation environments in northern Trinidad. This system has been studied extensively at the phenotypic level, but little is known about the underlying genetic architecture. Here, we use four independent F2 QTL crosses to examine the genetic basis of seven (five female, two male) guppy life history phenotypes and discuss how these genetic architectures may facilitate or constrain rapid adaptation and convergence. We use RAD-sequencing data (16,539 SNPs) from 370 male and 267 female F2 individuals. We perform linkage mapping, estimates of genome-wide and per-chromosome heritability (multi-locus associations), and QTL mapping (single-locus associations). Our results are consistent with architectures of many loci of small-effect for male age and size at maturity and female interbrood period. Male trait associations are clustered on specific chromosomes, but female interbrood period exhibits a weak genome-wide signal suggesting a potentially highly polygenic component. Offspring weight and female size at maturity are also associated with a single significant QTL each. These results suggest rapid, repeatable phenotypic evolution of guppies may be facilitated by polygenic trait architectures, but subsequent genetic redundancy may limit gene re-use across populations, in agreement with an absence of strong signatures of genetic convergence from recent analyses of wild guppies.

## Introduction

Recent evidence that phenotypes can evolve rapidly (Sanderson et al. 2021) and often with surprising repeatability (convergence) (Waters and McCulloch 2021) has led to a re-evaluation of our expectations surrounding adaptation in nature. Particularly, understanding the genetic architecture of traits associated with both rapid adaptation and convergence is needed to understand how adaptive variation may be maintained and be made available to respond to sudden changes in selection. Alongside trait heritability, the architecture of adaptive traits is predicted to moderate dependencies on the initial frequency of potentially adaptive alleles, as well as the size of the phenotypic response to selection, ultimately impacting population viability (Kardos and Luikart 2021). Empirical support for such predictions is important due to the current global circumstances of rapid environmental change, and to understand adaptation more generally. Quantitative traits can have architectures made up of many loci of small-effect (polygenic), single (monogenic), or few (oligogenic) loci of large-effect, or a combination of these. There are currently theoretical expectations surrounding which of these are most likely to underlie rapidly evolving (Pritchard et al. 2010; Jain and Stephan 2017b) and/or convergent phenotypes (Yeaman et al. 2018) but empirical evidence supporting these hypotheses is only starting to accumulate. Here we aim to elucidate the genetic architecture of a canonical example of convergent and rapid evolution using a quantitative genetics approach: life history traits in Trinidadian guppies.

Knowledge of the genetic architecture of rapidly evolving traits is needed to test theoretical predictions. Polygenic traits may facilitate rapid adaptation by providing a substrate of standing genetic variation (SGV) to be exploited (Jain and Stephan 2015, 2017a; Barghi et al. 2019), enabling populations to adapt to shifting fitness optima by many small changes (Jain and Stephan 2017b). Indeed, Fisher’s fundamental theorem states that the rate change of mean fitness is equal to the amount of additive genetic variance for fitness (Fisher 1930; Stephan 2021). Furthermore, small-effect loci that are already observed at appreciable frequencies as SGV, due to mutational-input, balancing and temporal selection, and migration (Barton and Keightley 2002), are less likely to be lost through drift compared with new mutations of equivalent effect size (Dittmar et al. 2016). Thus, the extent of drift in small populations may limit rapid polygenic adaptation, particularly for loci at low frequency (John and Stephan 2020). Recent examples of suspected polygenic bases involved in rapid adaptation include shell morphologies of *Littorina* periwinkles (Westram et al. 2018), immunity phenotypes in response to myxomatosis in rabbits (Alves et al. 2019), and killifish adapting to anthropogenic thermal effluent runoff (Dayan et al. 2019).

Rapid adaptation of monogenic and oligogenic traits is expected to occur through selective sweeps, potentially leading to ‘overshooting’ of peaks across the fitness landscape incurring genetic load (Dittmar et al. 2016; Buffalo and Coop 2019). Whilst large-effect loci can be maintained as SGV, particularly with spatially varying selection and migration (Yeaman 2015), locally deleterious large-effect loci are expected to carry greater fitness costs, which may limit the abundance of large-effect SGV compared with small-effect SGV (Huang et al. 2016). Large-effect loci can also comprise units of many tightly linked small-effect loci, which may facilitate their maintenance within populations whilst responding to selection comparably to single large-effect loci (Oomen et al. 2020). Because large-effect loci allow for rapid transitions across fitness landscapes, including crossing low-fitness valleys, they may be particularly important for rapid responses to sudden, extreme, environmental change that affects absolute fitness (Collins and de Meaux 2009; Bell 2013; Whitehead et al. 2017). Taken together, rapid adaptation may be facilitated by either highly polygenic loci or loci of large-effect, depending on factors such as the maintenance of additive genetic variance or the severity of fitness costs. Knowledge of the underlying genetic architecture within rapidly adapting systems may therefore be informative of these underlying mechanisms.

Genetic architecture will also play a role in whether phenotypic convergence equates to genetic convergence. For example, if new optima are close enough that they can be reached by a subset of available SGV (Dittmar et al. 2016; Barghi et al. 2020), polygenic architecture reduces repeatability by increasing redundancy in the mapping of genotype to phenotype (Yeaman et al. 2018; Barghi et al. 2020; Láruson et al. 2020). In contrast, if genetic architectures are composed of few large-effect loci, present within populations at appreciable frequencies (Barton and Keightley 2002), reduced redundancy can funnel adaptation through repeatable genetic paths. Several notable examples of genetic convergence are single loci of large-effect (Stern 2013), including the *eda* gene associated with marine-freshwater armour plate phenotypes in three-spined stickleback (Colosimo 2005), and the *optix* gene associated with wing patterning across *Heliconius* species (Reed et al. 2011).

The guppies of northern Trinidad have provided compelling phenotypic empirical evidence for both rapid adaptation and convergent evolution. In this system, barrier waterfalls within rivers create replicated downstream/high-predation (HP) and upstream/low-predation (LP) habitats, with LP-adapted guppies evolving independently from HP sources. HP and LP sites within rivers exhibit contrasting levels of gene flow (Whiting et al. 2021), which if strong enough may predict architectures of tightly linked clusters of adaptive loci (Yeaman and Whitlock 2011; Yeaman 2013). LP populations are typically longer-lived, and exhibit larger adult sizes, reduced brood size, longer time to reach maturity and longer interbrood periods than their HP counterparts (Reznick 1982; Reznick et al. 2001; Torres Dowdall et al. 2012). LP life history phenotypes evolve rapidly following experimental translocations of HP guppies (Endler 1980; Reznick and Bryga 1987; Reznick et al. 1990, 1997, 2019; Gordon et al. 2009). Guppies raised under laboratory conditions for multiple generations continue to exhibit differences between HP and LP phenotypes, indicating traits have a heritable genetic basis (Reznick 1982; Torres Dowdall et al. 2012). Little is known, however, about the genetic architecture of these traits. Here we present results for the first quantitative genetics mapping cross in this system for life history traits; crossing two genetically and phenotypically divergent populations in an F2 design to maximise the genetic resolution (i.e., maximise informative markers) and phenotypic variance of traits within the F2 population.

Life history traits are commonly involved in adaptation to novel or changing environments, but exhibit a range of architectures (Oomen et al. 2020). These include highly polygenic, classically quantitative traits, such as clutch size and egg mass in great tits (Santure et al. 2013) and weight of Soay sheep (Bérénos et al. 2015), but also traits with single loci explaining a large proportion of phenotypic variance, such as age at maturity in Atlantic salmon (*Salmo salar* L) (Ayllon et al. 2015; Barson et al. 2015) and other salmonids (Moghadam et al. 2007; Kodama et al. 2018), and growth rate in common frogs (Palomar et al. 2019).

Sex differences between life history traits have also been observed. In platyfish (live-bearers, like guppies), male size at maturity is linked to a large-effect locus, where non-signal transducing copies of *mc4r* genes in large males act as a dominant mutation to delay the onset of maturity (Lampert et al. 2010). In live-bearers, males stop growing at maturity, so size is linked to the onset of maturity, while females grow after maturity (Reznick and Endler 1982). Similar sex differences have been observed in salmon, where the *vgll3* locus is predictive of male, but not female, age at maturity (Ayllon et al. 2015).

Mapping large-effect loci is often achieved with quantitative trait locus (QTL) crosses or genome-wide association studies. However, these approaches can inflate the prominence of individual loci and have come under scrutiny (Rockman 2012; Slate 2013). Indeed, multi-locus analyses have resolved some empirical inconsistencies, such as the ‘missing heritability crisis’ (Manolio et al. 2009) for classic quantitative traits such as human height (Yang et al. 2010, 2011b); bringing empirical evidence back in line with Fisher’s prediction of the ‘infinitesimal model’ (Fisher 1918; Barton et al. 2017).

Using an F2 QTL breeding cross design, we examine the genetic basis of seven life history traits in guppies: female age (1) and size (2) at first brood, first brood size (3), interbrood period (4), average dry offspring weight in the first brood (5), and male age (6) and size (7) at maturity. Our aims are to assess the relative extent to which different facets of life history have significant genetic bases, and whether guppy life history traits are better explained by polygenic, or monogenic/oligogenic models. By exploring the genetic bases of these traits, we can begin to understand the role that their genetic architecture has played in their rapid and convergent evolution in the wild.

## Materials and methods

### Crosses

**Fig. 1:**
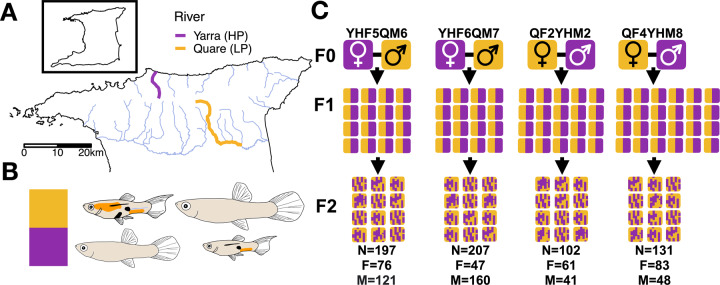
Sampling rivers in Trinidad and cross design. Sampling rivers are highlighted along with a number of major rivers from northern Trinidad’s three drainages (**A**). Males (smaller, colourful) and females (larger, uncoloured) in panel **B** highlight common morphological differences between HP (smaller, less colourful) and LP individuals. Crossing design for the four families is shown in panel **C**, illustrating two crosses in both directions, the number of F1 parents (crossed in pairs), and the final numbers of F2 individuals in all families.

Fish were second- and third-generation laboratory-reared individuals from an HP site in the Yarra river (680415E, 1193791N) and an LP site in the Quare river (696907E, 1181003N) (Fig. 1A). These populations have demonstrable HP-LP life history phenotypes (Supplementary Table S1, Fig. 1B, and Supplementary Fig. S1), and have been studied extensively in prior work (Reznick 1982; Reznick et al. 1996, 2004, 2005). Four F2 full-sib intercrosses were performed (Fig. 1C). Two crosses were performed for each cross direction in which wild-caught LP males were crossed with wild-caught HP females and vice versa. F1s were mated within cross and F2s were phenotyped and genotyped. Grandparents were also genotyped.

### Phenotyping and phenotype GLMs

Life history phenotyping and rearing followed (Reznick 1982); full rearing details are available in the Supplementary Material. The size of females and males was measured under a dissecting scope with Vernier callipers following MS-222 anaesthetisation. Based on the allometric dependency of female brood size, we took residual brood size as the difference between observed and linear-predicted brood size based on size. Male age at maturity was judged from the development of the apical hook. Interbrood period was scored as days between first and second parturition. Offspring weight was recorded as the mean dry weight of individuals from the first litter (data from the second litter was highly correlated). Where necessary, phenotypes were log-transformed to improve fit for normality assumptions. Details on rearing temperature recording can be found in the Supplementary Methods.

Rearing (mean temperature and date of birth (DOB)) and family effects on phenotypes were explored using generalised linear models (GLM) in R (v4 (R Core Team 2020)). DOB was included as a proxy for subtle unmeasured changes in rearing conditions over time. We performed backwards model selection using *step()* from a full additive model including family, temperature, and DOB. Relevant model assumptions were checked by comparing residuals to simulated residuals in the R package *DHARMa* (Hartig 2020). The final model term significance was determined based on ΔAIC and *F*-tests between models with and without each variable. Non-parametric Spearman’s rank correlations were used to confirm model effects if assumptions were violated. Adjusted partial *R*-squared was estimated for all final model variables with the R package *rsq* (Zhang 2020) using the variance-function-based type.

### Genotyping

Genomic DNA was extracted from fin clips using an ammonium acetate extraction method (Nicholls et al. 2000; Richardson et al. 2001). We genotyped each individual using a RAD-sequencing library preparation method adapted from Poland et al. (Miller et al. 2007; Baird et al. 2008; Poland et al. 2012); full genotyping details are available in the Supplementary Material. Of all 661 individuals used in the final analysis, 61 were sequenced two or three times more to account for low coverage (‘merged’ individuals in Supplementary Table S2). To ensure optimal coverage, and reduce PCR duplicate effects, all eight grandparents were sequenced four times in four separate PCR reactions and sequencing libraries. Of the total 653 F2s, 637 (370 males, 267 females) were used for phenotype analyses due to missing phenotypes for 16 individuals.

### Bioinformatic processing

Raw read data were trimmed and adaptors removed using cutadapt (Martin 2011). Stacks v2.5 was used for all downstream processing (Rochette et al. 2019). Trimmed read data were used as input to process_radtags, with options to remove reads with uncalled bases (-c), quality filter at Q10 (-q), and rescue barcodes and RAD tags containing sequencing error (-r). Cleaned RAD tags were aligned to the male guppy reference genome (Fraser et al. 2020) using BWA-MEM (Li 2013), and converted to bam format using samtools. Read group information was added to bam files using Picard Tools v2.6.0 AddOrReplaceReadGroups (Broad Institute 2019) and alignments of the same individual were merged using Picard Tools MergeSamFiles. Bam files were used as input to the gstacks module in Stacks2, retaining alignments with a minimum mapping quality of 20. The final VCF contained only loci called across all individuals (-p 1), at a max-missing frequency of 80% (-r 80) and minor allele frequency of 5% (-maf 0.05). Samples were retained with ≥15× average coverage, average coverage across the samples was 33.4× (Supplementary Table S2) and average missing data was 0.05%. For QTL scans, linkage mapping was performed using Lep-MAP3 (Rastas 2017) (see Supplementary Methods for full details) and genotypes were imputed based on grandparental phasing during linkage mapping.

### Heritability and multi-locus estimates of trait architecture

We used genome-wide complex trait analysis (GCTA) (Yang et al. 2011a) to estimate phenotype heritability. This approach estimates the heritability of phenotypes by partitioning phenotypic variance into genetic variance (specifically of the SNPs sequenced rather than heritability in the traditional sense), random genetic effects and residual variance using the restricted maximum likelihood method within a linear mixed model. Heritability estimates using this method are comparable to true pedigree studies (Stanton-Geddes et al. 2013; Duntsch et al. 2020). We estimated a genetic relatedness matrix (GRM) using all SNPs for males and females separately. SNPs from scaffolds were merged onto the beginning/end of chromosomes according to the linkage map (Supplementary Table S3) to improve the accuracy of per-chromosome estimates. We included the first three principal components (which separated the four families) as quantitative covariates, allowing for among-family intercepts to vary. Rearing covariates were included in a trait-specific manner if these were associated with the phenotype (Supplementary Table S4). To assess within-family effects, we included an additional GRM calculated with the *--make-bK 0.05* parameter, allowing us to partition variance into the sequenced SNPs (V_G1_) and within-family structure (V_G2_). At the per-chromosome level, including this additional GRM prevented model convergence in some cases (11/138 chromosome-phenotype pairs), but we observed negligible differences in heritability estimated with and without this additional GRM at the whole-genome level.

To estimate the heritability associated with specific chromosomes (*h*^*2*^*c*), we took two approaches: (1) we partitioned phenotypic variance using a model with a GRM derived from the focal chromosome and quantitative covariates; (2) we used a likelihood-ratio test (LRT) approach following Santure et al. (2013, 2015) and Duntsch et al. (2020) in which we compared a full model *a* (GRM based on all chromosomes except focal chromosome + focal chromosome GRM) to a reduced model *b* (without focal chromosome GRM). Quantitative covariates were included in both models. Models were compared with a LRT according to (1), with *p* values taken from the *χ*^2^ distribution with one degree of freedom.

1 $${\rm{LRT}} = - 2\left( {L_a - L_b} \right)$$

Correlation between chromosome size and heritability can reveal highly polygenic architectures, assuming chromosome size is a proxy for functional loci count. Previous work suggests *p* values derived from both chromosome partitioning approaches highlighted above are comparable (Kemppainen and Husby 2018a), so we used *h*^*2*^*c* estimations from focal chromosome GRMs (approach 1) due to issues with model convergence at low *h*^*2*^*c* under approach 2 (see Supplementary Table S5). We corrected for heteroscedasticity among chromosomes and the non-negative nature of *h*^*2*^*c* using the *HC_Correction()* function (Kemppainen and Husby 2018b).

### QTL scans

We produced two datasets for QTL-scanning. The first included fully informative SNPs for both founding populations, i.e., SNPs that were homozygous in all eight grandparents and fixed for alternative variants between HP- and LP-derived grandparents. This dataset included 1220 SNPs (of the total 6765 markers in the linkage map, see below), and allowed for analysis of all individuals together to increase biological power, where individuals could inherit an HP (H) or LP (L) allele. The second dataset comprised four separate datasets, one for each family in which family-informative SNPs were included for each family (homozygous, alternative, SNPs in each grandparent within families). Numbers of family-informative SNPs for each family were similar (3436; 3400; 3476; and 3476 for QF2YHM2, QF4YHM8, YHF5QM6, and YHF6QM7, respectively). These datasets provided increased SNP resolution within families, and allowed us to examine alleles that may have only been segregating in single crosses. These latter datasets cannot be used to assess loci that are Y-linked, because all males within a family carry the same Y, and the effect of different Y-loci among families cannot be separated from general genome-wide relatedness.

We first performed single-locus scans using the *scan1()* function in R/qtl2 (Broman et al. 2019). We inserted pseudomarkers using *insert_pseudomarkers()* with step = 1, calculated genotype probabilities with *calc_genoprob()* and an error_prob = 0.002, and converted genotype probabilities to allele probabilities using *genoprob_to_alleleprob()*. We calculated a grid with *calc_grid()*, subsetted genotype probabilities to grid pseudomarkers with *probs_to_grid()*, and used this to calculate a kinship matrix with *calc_kinship()* according to the leave-one-chromosome-out method. Rearing covariates and a binary family assignment matrix were included as additive covariates. The significance of LOD peaks was determined by 1000 permutations for all models with *operm()* at an α of 0.05. For scans of the sex-determining region, inputs were merged for males and females, and the same methodology was used, with the exception that sex was modelled as a binary phenotype, and rearing covariates were excluded from the covariate matrix.

We also explored QTL scans that allow for multiple QTLs using the *stepwiseqtl()* function in R/qtl (Broman et al. 2003) which is not available in R/qtl2. This approach assesses interactions among all pairs of loci, allowing for epistatic effects to be examined. We allowed for models with a maximum of six loci, used the imputation method, and allowed for only additive interactions among loci. The significance of LOD peaks was determined based on 1000 permutations.

## Results

### Phenotypes

Both male age (GLM: *F*_3,340_ = 14.75, *p* = 4.82e^−9^) and size at maturity (GLM: *F*_3,340_ = 16.02, *p* = 9.30e^−10^) of F2s varied significantly between the four cross families (Supplementary Fig. S2A and Supplementary Table S4). For age at maturity, cross YHF5QM6 F2s tended to mature later than all other crosses (Tukey *p* < 0.05), and for size at maturity, cross QF4YHM8 F2s matured at a larger size. Rearing conditions affected both male phenotypes. Males matured earlier under increased temperatures (GLM: *F*_1,340_ = 13.75, *p* = 2.40e^−4^) and if born later in the experiment (GLM: *F*_1,340_ = 23.26, *p* = 2.15e^−6^). Larger males at maturity tended to be born later in the experiment (GLM: *F*_1,340_ = 41.51, *p* = 4.06e^−10^), but the temperature did not affect size at maturity. Family status explained 10.4 and 11.7% of phenotypic variance for age and size at maturity, respectively, an effect that will not be captured by our subsequent mapping approaches. Male age and size at maturity were not strongly associated with one another (correlation of individuals, Spearman’s ρ = −0.07, *p* = 0.184), but a GLM of male size at maturity predicted by an interaction between age at maturity and family revealed a significant effect (GLM: *F*_3,340_ = 2.98, *p* = 0.031), with both positive and negative associations depending on the family.

Female phenotypes were generally less variable among families, only female size at first brood (GLM: *F*_1,265_ = 11.88, *p* = 6.04e^−4^) and offspring weight (GLM: *F*_1,249_ = 3.03, *p* = 0.030) differed significantly between families (Supplementary Fig. S2B and Supplementary Table S4), with the latter effect only marginally significant. Consistent with a general life history axis, covariance among female phenotypes was generally high (Supplementary Table S6). All female traits loaded positively onto PC1 (37.6%), with female age (loading = 0.68) and size (loading = 0.58) loading particularly strongly. PC2 (27.0%) explained residual variance associated with brood traits, with first brood size (−0.62) and interbrood (−0.59) loading negatively, and offspring weight loading positively (0.44). PC2 therefore summarises variation among females with few, heavier offspring and short interbrood periods, and vice versa. Similar to males, females reached maturity and produced their first brood earlier under increased temperatures (GLM: *F*_1,265_ = 21.65, *p* = 5.19e^−5^). Increased temperature also reduced interbrood period (GLM: *F*_1,265_ = 24.32, *p* = 1.45e^−6^) and females born later in the experiment had longer interbrood periods (GLM: *F*_1,265_ = 13.47, *p* = 2.94e^−4^). Other female phenotypes were not associated with rearing conditions (Supplementary Table S4). In contrast to male phenotypes, family status explained much less phenotypic variance: 6.7% for first brood size, 5% for size at first brood, and 2.2% for offspring weight (‘family’ was dropped from other models due to low explanatory power).

### Linkage mapping

The final linkage map consisted of 6765 markers and a length of 1673.8 cM. There was good overall concordance between this genetic map and the recently updated reference genome (Fraser et al. 2020), with the additional placement of unplaced scaffolds on all LGs. There were also minor structural rearrangements and inversions (Supplementary Fig. S3), for which corroborative support was found from previously published HiC data (Fraser et al. 2020). Typically, unplaced scaffolds were joined to either chromosome end (Supplementary Table S3 and Supplementary Fig. S3).

### Heritability and multi-locus estimates of trait architecture

Heritability varied between traits, but in almost all cases (excluding first brood size) the variance explained by within-family structure (V_G2_) was greater than the variance explained by the specific SNPs themselves (V_G1_). This is expected given RAD-sequencing is designed to capture SNPs in linkage with causal variants, rather than the causal variants themselves. Estimates of heritability were greatest for male size at maturity (43.4%), offspring weight (33.4%), male age at maturity (31.3%), and interbrood period (30.2%). Estimates for the remaining female life history traits were lower, not exceeding 9.7% (female size at first brood), with standard errors that overlapped 0 (Table 1). We repeated analyses of male trait heritability by averaging over 100 randomly downsampled datasets with the same number of males as females (*N* = 267) and comparable statistical power (Supplementary Fig. S4) (*N* = 200) (see Supplementary Table S7 and Supplementary Methods for details on power analyses). In both downsampled datasets we retained similar signatures of heritability for both male traits (Supplementary Table S8), demonstrating that the differences in heritability between male traits and female traits are unlikely due to differences in statistical power.

**Table 1:** Estimates of genome-wide heritability for each phenotype based on GCTA-GREML.

Phenotype	V_G1_	V_G2_	V_e_	V_P_	V_G1_/V_P_	V_G1+G2_/V_P_
Age at first brood (F)	0 ± 0	0 ± 0	0.02 ± 0	0.02 ± 0	0.03 ± 0.19	0.033 ± 0.1
Size at first brood (F)	0 ± 0	0 ± 0	0.01 ± 0	0.01 ± 0	0 ± 0.2	0.097 ± 0.11
First brood size (F)	0.78 ± 1.96	0.12 ± 2.14	8.86 ± 1.09	9.76 ± 0.89	0.08 ± 0.2	0.092 ± 0.1
Interbrood period (F)	0 ± 0	0 ± 0	0.01 ± 0	0.02 ± 0	0.17 ± 0.27	0.302 ± 0.12
First brood offspring weight (F)	0 ± 0.02	0.03 ± 0.03	0.06 ± 0.01	0.09 ± 0.01	0 ± 0.28	0.334 ± 0.12
Age at maturity (M)	0 ± 0	0.004 ± 0	0.009 ± 0	0.013 ± 0	0 ± 0.2	0.313 ± 0.09
Size at maturity (M)	0 ± 0.07	0.156 ± 0.08	0.203 ± 0.03	0.358 ± 0.03	0 ± 0.18	0.434 ± 0.09

**Fig. 2:**
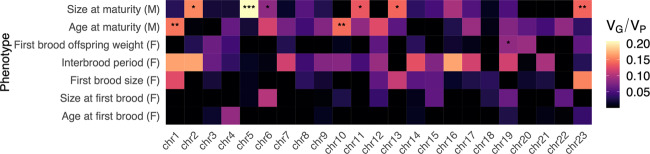
Estimates of phenotypic variance proportions explained by per-chromosome genetic relatedness matrices (*h*^*2*^*c*). Tiles are coloured according to the relative proportion of phenotypic variance (V_P_) explained by genetic variance (V_G_). FDR-corrected *p* values (corrected within phenotypes across all chromosomes) are displayed as asterisks *FDR ≤ 0.05; **FDR ≤ 0.01; ***FDR ≤ 0.001.

We repeated the analysis on each chromosome to test whether these estimates could be explained disproportionately by certain chromosomes, or whether per-chromosome associations may exist that cannot be observed within genome-wide estimates. Estimates of *h*^*2*^*c* based on single-chromosome GRMs revealed six chromosomes significantly associated with male size at maturity, four chromosomes with male age at maturity, and one chromosome with offspring weight (FDR ≤ 0.05; Supplementary Table S9 and Fig. 2A). Of these however, according to the LRT approach only three chromosomes for male size at maturity (chr5: 20.7%, chr23: 13.9%, chr11: 13.3%), two for male age at maturity (chr1: 14.7%, chr10: 14.4%) and one for offspring weight (chr19: 8.4%) were significantly associated (LRT *p* ≤ 0.05; Supplementary Table S5). Following multiple-testing correction within phenotypes, only the associations between chr5 and male size at maturity (LRT = 9.346, fdr = 0.046), and chr19 and offspring weight (LRT = 9.264, fdr = 0.046) were significantly associated according to both methods. Agreement between both methods was good according to a correlation of *p* values (Spearman’s ρ = 0.827, *p* < 2.2e–16). Whilst this correlation is strong, there is a clear downward biasing of *p* values from single-chromosome GRMs, evident as a shift away from the y=x relationship (Supplementary Fig. S5).

We found positive correlations associated with single-chromosome GRM estimates of *h*^*2*^*c* and chromosome size (following the addition of scaffold sizes according to the linkage map; Supplementary Fig. S6) for interbrood period (*r* = 0.373, *p* = 0.04, HC-corrected *p* = 0.105) and male size at maturity (*r* = 0.253, *p* = 0.122, HC-corrected *p* = 0.231); however, these were not significant following HC-correction. Other traits exhibited negative and non-significant correlations, and in some cases, correlations were clearly driven by one or a few chromosomes, rather than describing a wider whole-genome effect (Supplementary Fig. S6).

### QTL mapping

We first mapped sex as a binary trait. The location of the sex-determining locus has been narrowed down to a small region at the distal end of chromosome 12 (Fraser et al. 2020; Charlesworth et al. 2020a). Our QTL mapping recovered a single large peak on chromosome 12 (17.79 cM), with confidence intervals extending from 5.35 to 27.78 cM (Supplementary Fig. S7). In our map, the region following this (27.78–61.79 cM) corresponds to the very distal tip of chromosome 12 (approximately 24.6 Mb onwards, plus additionally placed scaffolds), which is the only fully recombining region of this chromosome and is pseudoautosomal (Charlesworth et al. 2020b). This places the sex-determining region somewhere in the non-recombining region immediately prior to the pseudoautosomal region, as proposed by others (Fraser et al. 2020; Charlesworth et al. 2020a). This analysis therefore confirms good power to detect loci of large-effect and confirms previously published information about the sex chromosome and region containing the sex-determining locus.

**Fig. 3:**
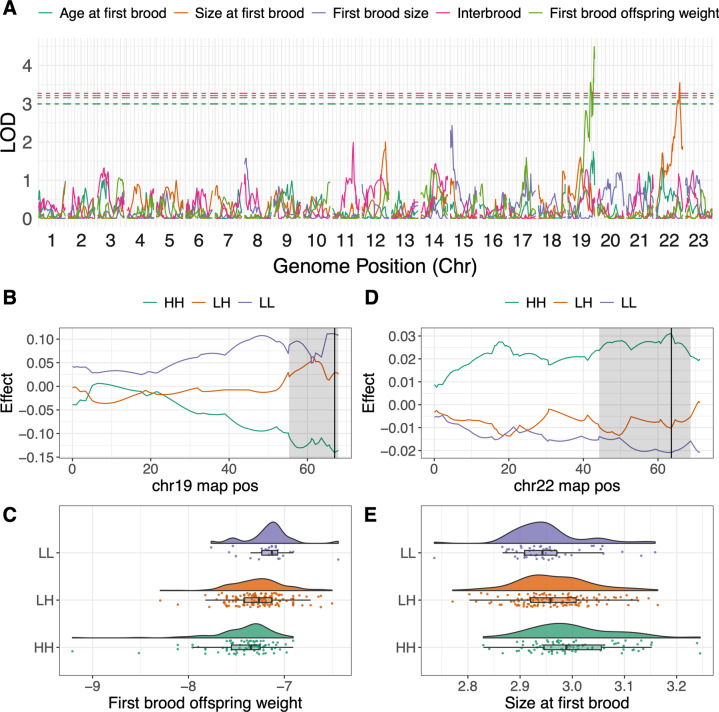
Single-locus QTL scans for female life history traits: age at first brood (days), size at first brood (cm), first brood size (residual), interbrood period (days) and first brood offspring weight (g). All female traits were log-transformed. **A** Genome-wide additive model scan results for all traits, 5% significance thresholds are denoted. Significant QTLs for offspring weight (chr19, **B**, **C**) and size at first brood (chr22, **D**, **E**) are visualised in further detail. **B–E** QTL effects across the focal linkage groups (**B** and **D**), and distributions of phenotypes across genotypes at the peak (**C** and **E**). In **B** and **D**, the QTL peak is shown as a black line, with confidence intervals (LOD drop = 1.5) highlighted by grey shaded areas.

We then mapped female traits using all fully informative markers (*N* = 1220, median distance between markers = 140.293 kb or 0.536 cM, median markers per chromosome = 50, min = 33 [chr7], max = 82) across all 267 females. Applying a permuted 5% threshold (*N* = 1000), we detected two QTLs (Table 2). The strongest QTL was associated with first brood offspring weight at the very distal tip of chr19 (chr19:66.954) (Fig. 3B), explained 7.46% of phenotypic variance, and exhibited additive effects in which the HH homozygotes produced smaller offspring than LL homozygotes (Fig. 3C). Confidence intervals (drop in LOD of 1.5) extended between 55.335–67.877 cM. The other QTL was associated with size at first brood (chr22:63.593) (Fig. 3D), explained 5.94% of phenotypic variance, and exhibited additive effects in which the HH homozygotes had their first brood at a larger size than LL homozygotes (Fig. 3E); contrary to HP-LP expectations. Confidence intervals for the chromosome 22 QTL extended between 44.235 and 68.753 cM.

**Table 2:** Summary of all whole-dataset and within-family large-effect QTL associated with guppy life history identified in this study.

Dataset	Phenotype	QTL (marker ± 1.5 LOD drop)	LOD	PVE (%)	QTL effect^a^
All females	Offspring weight	chr19:66.954 (55.335–67.877)	4.493	7.46	**L** > **H**
All females	Size at first brood	chr22:63.593 (44.235–68.753)	3.552	5.94	H > L
YHF5QM6 (females)	First brood size	chr23:37.33 (4.589–53.588)	3.386	18.55	**H** > **L**
YHF5QM6 (females)	Interbrood	chr12:60.713 (54.701–72.487)	3.406	18.65	H > L
QF4YHM8 (females)	Interbrood	chr14:28.41 (28.412–67.316)	3.559	17.90	**L** > **H**
YHF6QM7 (males)	Length at maturity	chr23:31.741 (0–52.193)	3.462	9.48	**L** > **H**

^a^Values in bold are in the expected direction based on natural populations.

Scans within each of the four families (see Supplementary Table S10 for marker numbers and distances) identified an additional three QTLs (Table 2 and Supplementary Figs. S8 and S9). Two of these were observed in cross YHF5QM6, associated with first brood size (chr23:37.33, LOD = 3.386, ci low = 4.589 cM, ci high = 53.588 cM) and interbrood period (chr12:60.713, LOD = 3.406, ci low = 54.701 cM, ci high = 72.487 cM). These QTLs explained 18.55 and 18.65% of phenotypic variance within their families, respectively. The QTL linked with interbrood period is particularly interesting given its proximity to the sex-determining region. We also detected a QTL associated with female interbrood period in cross QF4YHM8 (chr14:28.41, LOD = 3.559, ci low = 28.412 cM, ci high = 67.316 cM), explaining 17.9% of phenotypic variance for this trait in this family.

**Fig. 4:**
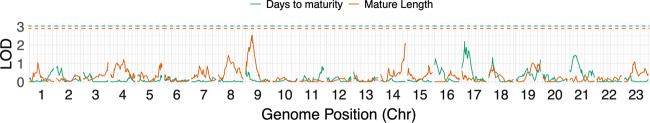
Single-locus QTL scans for male life history traits: days to maturity (log-transformed), length at maturity (mm). No significant QTLs were detected.

We applied the same methodology to male traits; however, we did not recover any significant QTLs across the whole-dataset (Fig. 4). Within the YHF6QM7 cross, we observed a significant QTL associated with male mature length on chromosome 23 (chr23:31.741, LOD = 3.462, ci low = 0 cM, ci high = 52.193 cM) (Table 2 and Supplementary Figs. S10 and S11). This confidence interval covered the majority of the chromosome, and the QTL explained 9.48% of phenotypic variance in this cross. At this QTL, HH homozygotes matured at a smaller size than LL homozygotes.

We also explored multi-QTL models using r/qtl’s *stepwiseqtl()*, allowing for models that fit up to six loci, with the same additive covariance matrices of family and rearing covariates used previously. For female models, this approach returned null models for all phenotypes except size at first brood and offspring weight, for which we recovered single QTL models including only the QTL loci previously identified above. For males, null models were returned for both phenotypes.

Expectedly, due to sample size differences, male QTL scans had greater statistical power than female QTL scans (Supplementary Fig. S4). However, given we only observed whole-dataset QTLs in the female dataset; a difference in the statistical power between males and females does not explain the absence of signatures of large-effect loci in males compared with females.

### Candidate genes

Taken together, we find that where traits are heritable they are associated with whole chromosomes, suggestive of large polygenic regions, rather than single large-effect QTLs. However, we observed two significant QTLs for female traits (offspring weight chr19:66.954 and size at first brood chr22:63.593). We also observe several significant QTLs within specific families. Of these, the QTL associated with interbrood period in cross YHF5QM6 (YHF5QM6-chr12:60.71) is of particular interest given its proximity to the sex-determining region and relatively narrow confidence intervals. We therefore explored these three regions further for candidate genes.

The QTL at chr19:66.954, associated with offspring weight, covered ~8.2 Mb of chromosome 19 and included 267 genes, with 40 genes within 0.5 Mb of the QTL peak (chr19:18602889-19602889). The chr22 QTL peak (for size at first brood) was on scaffold 000111F_0:651336 with confidence intervals extended over the scaffold (00111F_0:528180-1199617) and the distal end of chromosome 22 (chr22:23415429-24223839), covering 45 genes. The interbrood period QTL on chr12 overlapped the YTH domain containing 1 gene, *ythdc1*, on scaffold 000149F_0 (000149F_0:131600). This scaffold was placed at the distal end of chromosome 12 near the sex-determining region, in agreement with other mapping studies (Charlesworth et al. 2020a), and corresponds with scaffold KK215301.1 in the older genome assembly (Künstner et al. 2016). The confidence interval around this QTL covered additional regions of scaffold 000149F_0 (000149F_0:47124-197258) and chromosome 12 (chr12:24525856-24705290) and included 18 genes. The *ythdc1* gene is a particularly promising candidate for female interbrood period given functional evidence for a role in oocyte development in mice (Kasowitz et al. 2018) and zebrafish (Xia et al. 2018). For a full description of genes in each QTL region, and additional analysis of the *ythdc1* gene, see the Supplementary Materials and Supplementary Tables S11 and S12.

## Discussion

Using an F2 cross design of outbred populations with divergent life history phenotypes, we have demonstrated evidence of both small-effect (polygenic) and large-effect (oligogenic/monogenic) trait architectures underlying guppy life history evolution. Both male size and age at maturity, exhibit significant heritability associated with particular chromosomes, suggesting polygenic traits, but little evidence of genome-wide polygenicity or large-effect loci. This suggests these traits are underwritten by many small-effect loci on specific chromosomes, which may be clustered together. For the five female traits, we recovered significant genome-wide heritability for interbrood period and offspring weight. For offspring weight, all per-chromosome estimates of heritability were generally weak, but a large-effect locus detected on chromosome 19 may represent a significant proportion of genome-wide heritability. For interbrood period, we detected a weak genome-wide polygenic signal as an association between chromosome size and per-chromosome heritability and found evidence of a large-effect locus on the sex chromosome, LG12, in a single family. We also detected a significant large-effect QTL associated with female size at first brood with a small effect size, but negligible estimates of genome-wide heritability for this trait. Finally, we found no evidence for heritable genetic architectures for female first brood size and age at first brood, although we observed a within-family QTL associated with first brood size on chr23.

We applied genome-wide and chromosome-scale heritability analyses with QTL scans to rule out some uncertainty. For example, estimates of heritability in the absence of a QTL are most likely due to small-effect loci. Furthermore, a weak genome-wide signal of heritability coupled with detecting an individual QTL is likely due to a monogenic/oligogenic architecture. It is more difficult however at our level of sequencing resolution to distinguish between a QTL made up of individual loci of large-effect, or tightly linked clusters of small-effect loci. In terms of response to selection, however, a tightly clustered collection of small-effect loci may carry more comparable expectations to a large-effect allele than a disparate assortment of small-effect loci (Oomen et al. 2020), depending on the extent of linkage within the QTL; as observed recently in three-spined stickleback rapid adaptation (Roberts Kingman et al. 2021).

The significant genetic components of guppy life history phenotypes found here are in line with previous laboratory rearing estimates of heritability. Specifically, the high heritability of male traits, interbrood period, and offspring weight has been documented in laboratory-reared populations under controlled conditions (Reznick 1982) and in LP-introduction experiments (Reznick et al. 1997). Consistent differences between laboratory-reared HP and LP populations for female size and age at maturity and interbrood period have been observed (Reznick 1982), with similar results reported along a predation gradient in wild-caught vs laboratory-reared guppies from the Guanapo river (Torres Dowdall et al. 2012). However, female age and size at maturity from experimental LP introductions have shown inconsistent, negligible, estimates of heritability (Reznick et al. 1997). This latter study also postulates that the higher heritability, and more rapid evolution, of male traits may involve significant Y-linked loci, but we found no evidence to support this (although see discussion on Y-linked traits below). Rather, our results of a significant polygenic component of male age and size at maturity also predict more rapid phenotypic evolution for males through increased SGV.

Recent work has sought to compare the relative contributions of genetic and plastic effects on guppy life history. HP-LP comparisons of wild-caught fish are confounded by there being higher guppy population densities and lower light availability (and primary productivity) in LP sites (Reznick et al. 2001). Common garden experiments performed on second-generation, laboratory-reared guppies reveal no interaction between food availability and HP-LP life history differences (Reznick 1982; Reznick and Bryga 1996), so there are strong phenotypic responses to food availability, but no evidence of adaptation to food availability per se. Felmy et al. (2022) demonstrated that guppy life histories cluster together in terms of those strongly affected by food-induced plasticity (predominantly size-related traits), those affected by HP-LP ecotype (including interbrood interval and offspring weight), and those affected by both or neither. For female traits, these observations align well with our results, particularly the higher heritability for interbrood period and offspring weight (Table 1), and the absence of strong signatures of genetic architectures associated with female size at maturity. Felmy et al. (2022) also demonstrate food-induced plasticity for male age and size at maturity; however, these likely operate alongside underlying genetics, in agreement with observations here, and documented in other studies of male guppy life history (Reznick 1982; Reznick et al. 1997, 2005). Furthermore, resource-associated plasticity explains comparable phenotypic variance to heritability for female reproductive investment (Reznick and Yang 1993). Predator cues are an additional source of plasticity, affecting female size at maturity (Torres-Dowdall et al. 2012), and growth rate (Handelsman et al. 2013). Here, we found male age and size at maturity and female age at first brood and interbrood period exhibited plasticity linked to rearing conditions, reflecting small fluctuations in temperature (between 23.3 and 27.1 °C) and DOB (a proxy for unmeasured conditions). Whilst we included rearing effects and family classification as covariates in our models, and controlled for resources during rearing, it remains possible that additional sources of plasticity may have obscured our analyses.

A limitation of our crossing design is that we cannot make across-family comparisons. We controlled for family-specific intercepts in models, either by including kinship information or family status as covariates. However, family status accounted for significant phenotypic variance in five of the seven phenotypes (all except interbrood period and female age at first brood; Supplementary Table S4). These may be attributed to family-specific alleles segregating across the genome, or Y-linked loci specifically; however, we cannot separate these within the current dataset. We identified some large-effect loci segregating within families (Supplementary Figs. S8–S11); however, our mapping of male phenotypes in particular will be blind to Y-specific QTLs, given all males within a family share the same Y allele. Y-linked loci have been suggested to affect age and size at maturity phenotypes in other poecilids (Kallman and Borkoski 1978; Lampert et al. 2010), so these regions may be important and comprise some of the phenotypic variance associated with family status (which is an upper limit).

Whilst we detected both polygenic and large-effect architectures for life history phenotypes, our study is limited by sample size. In part, this is due to the modest brood sizes of guppies, which restricted larger F2 families, and our focus on sex-specific phenotypes. It is well known within quantitative genetics studies that small sample sizes can inflate estimates of heritability and QTL effect sizes, termed the ‘Beavis Effect’ (Beavis 1994; Rockman 2012; Slate 2013). We therefore expect our QTL PVE estimates are upwardly biased, particularly within families. In particular, a small sample size inflates *p* values when run with single-chromosome GRMs with GCTA (Kemppainen and Husby 2018a), which likely explains why our LRT approach produced more modest *p* values (Supplementary Fig. S5). Our estimates of per-chromosome (*h*^*2*^*c*) and genome-wide heritability should therefore be treated with caution. Our combining of single-chromosome GRMs with a LRT approach however may be useful more generally to alleviate reciprocal issues of inflated *p* values with single-chromosome GRMs, and model convergence for chromosomes with minimal effects under the LRT approach.

The two main QTLs observed here, detected across the whole-dataset, reflect only moderate PVE (7.46% for offspring weight on chr19, and 5.94% for female size at first brood on chr22). Interestingly, each of these chromosome-phenotype pairings was also detected (marginal significance) by our multi-locus approaches. This suggests that these regions may be reasonably large, such that the peak (from which the PVE is calculated) only represents a portion of the variance explained by the wider region. Neither of these regions has been strongly implicated in HP-LP adaptation in previous population genomic analyses (Fraser et al. 2015; Whiting et al. 2021); however, Whiting et al. (2021) recorded a selection scan outlier within the chr22 QTL interval (chr22:23960000-23970000) in HP-LP comparisons from the Aripo and Madamas rivers. The closest gene to this outlier is *sox11a*. While selection scans of HP-LP comparisons cannot determine which phenotype selection might be acting on, our results here suggest this region may be involved in female growth. We also observed a number of within-family QTLs, which most likely represent segregating variation within populations (Table 2). In some cases, the direction of effect was the opposite of HP-LP expectations, which might indicate that these QTLs do not contribute towards phenotypic divergence between the source populations. These QTLs could, however, represent HP/LP-adapted alleles from the contrasting, unsourced, HP/LP population within the same river. Either way, we expect that the identification of these QTLs, regardless of the direction of effect, is informative for understanding the distribution of fitness effects and maintenance of variation within populations associated with these phenotypes.

If new optima can be reached by a subset of available SGV, the absence of prominent large-effect loci observed here is expected to fuel redundancy and limit molecular convergence; allowing independent populations to use different sets of alleles to produce convergent phenotypes (Barghi et al. 2020). Limited genomic convergence has been observed in two independent evaluations of natural HP-LP guppy populations (Fraser et al. 2015; Whiting et al. 2021), but appears to be more pervasive in experimental translocations of HP guppies to uncolonised LP habitats than naturally colonised LP populations (Fraser et al. 2015). Part of this discrepancy can be explained by the concept of ‘adaptive architecture’, such that the convergent genetic basis of polygenic traits can be influenced by additional factors such as starting allele frequencies. Initial allele frequencies or the amount of SGV are likely to be similar when experimental populations are founded from the same population and/or lack founding bottlenecks, compared with naturally derived HP-LP pairs in different rivers. Empirical evidence for genetic convergence occurring with polygenic traits has been observed for male mating song traits in Hawaiian *Laupala* crickets (Blankers et al. 2019) and myxomatosis resistance in rabbits (Alves et al. 2019), suggesting that genetic architecture alone is not necessarily a constraint on genetic convergence.

Trait architectures can also be informative for understanding the mechanisms driving rapid adaptation. Guppies evolve rapidly over the course of a few generations when translocated to LP environments (Reznick and Bryga 1987; Reznick et al. 1997, 2019; Gordon et al. 2009) or following manipulation of predation (Reznick et al. 1990). Our findings here, of polygenic architectures linked to guppy life history, are in keeping with recent empirical (Barghi et al. 2019) and theoretical work (Bell 2013; Jain and Stephan 2017a) suggesting these can facilitate rapid adaptation. In this framework, many small-effect loci provide adaptive substrate within populations to rapidly respond to shifting optima. This is particularly true if distances to new fitness optima are small, such that environmental change is modest and fitness effects are relative and non-lethal (soft selection); the latter is likely the case for guppy life history traits under LP regimes. This model could therefore allow male life history traits to change rapidly through small changes at many loci without experiencing fitness costs associated with reduced absolute fitness of intermediate phenotypes. Additional segregating larger effect loci may also act in concert with compensatory changes at small-effect loci for female traits, so long as distances between fitness peaks are large enough. Alternatively, the large-effect QTL detected here may represent clusters of small-effect loci (Barton and Keightley 2002).

Our results provide regions of the genome and candidate genes to explore further. For instance, an appreciation that much of the genetic basis of guppy life history traits may be polygenic informs experimental and sampling designs for future evolutionary studies of this system. In particular, temporal sampling and quantifying genome-wide temporal autocovariances of allele frequencies offers a promising avenue for studying the role of polygenic architectures in rapid adaptation (Buffalo and Coop 2019). The genomic regions identified here may serve as focal regions in these studies. Whilst repeatability of individual genes may be limited by polygenic architectures, there is some evidence of functional convergence in guppies (Whiting et al. 2021), which may be better elucidated with focussed assessments of polygenic signals within the genome. An additional avenue of research might involve investigating the relationship between gene flow, genetic architecture, and repeatability. The rate of gene flow between HP and LP sites varies greatly between rivers (Barson et al. 2009; Willing et al. 2010; Whiting et al. 2021), and increased gene flow is predicted to promote clustering of adaptive alleles into large-effect loci (Yeaman and Whitlock 2011; Yeaman 2013). We found some evidence of within-family QTLs, and QTLs in the opposite direction than expected, which may be indicative of within-river HP-LP large-effect alleles. Future sampling could exploit the added dimension of gene flow by crossing between HP and LP populations from high- and low-gene flow river pairs, and examining the underlying genetic architectures. These results represent the first step in understanding the role of genetic architecture in the rapid and convergent evolution of guppy life history traits.

## Supplementary information

Supplementary methods, results and figures

Table S1

Table S2

Table S3

Table S4

Table S5

Table S6

Table S7

Table S8

Table S9

Table S10

Table S11

Table S12

## Data Availability

All sequencing read data are available from the ENA (Study accession: PRJEB48691). All scripts and other data associated with analysis are available on GitHub (github.com/JimWhiting91/guppy_LH_QTL) and are archived on Zenodo (10.5281/zenodo.5938562). The VCF, phenotypes, and linkage map are deposited with dryad (10.5061/dryad.w3r2280sk).
