# Supplementary material for: On the genetic architecture of rapidly adapting and convergent life history traits in guppies
Source: Heredity (Edinb). 2022 Mar 8;128(4):250–60. doi: 10.1038/s41437-022-00512-6 (PMC8986872; doi:10.1038/s41437-022-00512-6)
Supplement: Supplementary file 1 — Supplementary methods, results and figures [file 41437_2022_512_MOESM1_ESM.pdf]

# On the genetic architecture of rapidly adapting and convergent life history traits in guppies

## Supplementary materials

### Author list:

James R Whiting[1], Josephine R Paris[1], Paul J Parsons[1,2], Sophie Matthews[1], Yuridia Reynoso[4], Kimberly A. Hughes[3], David Reznick[4], Bonnie A Fraser[1]

### Contents:

- Supplementary Methods
- Supplementary Results
- Figure S1 - Phenotypes of previously studied wild/lab guppies
- Figure S2 - Phenotype distributions by cross family
- Figure S3 - Linkage map
- Figure S4 - Power analyses for GCTA and QTL analyses
- Figure S5 - Association between single GRM and LRT approach p-values
- Figure S6 - Correlations between $h^2c$ and chromosome size
- Figure S7 - QTL scan results for sex
- Figure S8 - Within-family single-locus QTL scans for female life history traits
- Figure S9 - QTL effect plots for within-family female traits
- Figure S10 - Within-family single-locus QTL scans for male life history traits
- Figure S11 - QTL effect plots for within-family male traits
- Table S6 - PCA results for female life history traits
- Table S12 - dN/dS results across Poeciliids for *ythdc1* candidate

Note: Tables S1-5,7-11 are available as external .xlsx files

## SUPPLEMENTARY METHODS:

### *Phenotyping and Rearing*

Life history phenotyping and rearing followed (Reznick 1982). Fish were reared from birth to 25 days in groups of 5 or 6 in two-gallon tanks, then sexed and isolated. They were then reared one per aquarium on quantified rations with opaque barriers between tanks. At first, males were checked weekly to evaluate the development of the anal fin, which was used as the index of maturity. They were then checked as often as daily as the fin approached full metamorphosis, as judged from the development of the apical hook and the extension of the hood, with maturity decided when the hood reached or extended beyond the tip of the fin. Females were mated weekly, beginning shortly after they entered the phase of quantified rations. We scored their age when they first gave birth. From then on, females were only mated the day after parturition when they are receptive to mating. All offspring were preserved on the day of birth and the female was preserved after the birth of the third litter. Tanks were cleaned every other week. Size of females and males was measured after they had been anaesthetized with MS-222, with length being measured under a dissecting scope with Vernier calipers. Based on the allometric dependency of female brood size, we looked at the residual brood size derived from comparing the observed brood size with the linear-predicted brood size based on female size at maturity. Where necessary, phenotypes were log-transformed to improve fit for normality assumptions. We measured temperature in tanks on the left, middle, and right of each row on each shelf in the guppy facility using Fisherbrand™ Traceable™ Digital Thermometers and roughly each week. The mean rearing temperature per F2 individual was then estimated by taking the mean temperature of the tank or closest tank over the individual's lifetime.

### *Genotyping*

Genomic DNA was extracted from fin clips using an ammonium acetate extraction method (Nicholls et al. 2000; Richardson et al. 2001). We genotyped each individual using a RAD-seq library preparation method adapted from Poland and colleagues (Poland et al. 2012; Miller et al. 2007; Baird et al. 2008). Briefly, double-digested genomic DNA (enzymes: *PstI* and *MseI*) was

annealed with cut-site specific sequencing adaptors bearing individual barcodes. Barcoded samples were amplified via PCR (12 cycles, with barcoded samples multiplexed). Each individual had a unique barcode, using the original barcodes from Poland et al. (2012). A total of 12 multiplexed libraries were sequenced. Of the 661 individuals used in the final analysis (including eight grandparents), 61 individuals were sequenced two or three times in separate libraries to account for low coverage ("merged" individuals in Table S2). To ensure optimal coverage of the grandparents, and to reduce the effects of PCR duplicates, each of the eight grandparents were sequenced four times in four separate PCR reactions and sequencing libraries. Of the total 653 F2s, 637 (370 males, 267 females) were used for phenotype analyses due to missing phenotype for 16 individuals.

***Linkage mapping***

Linkage maps were produced with Lep-MAP3 (Rastas, 2017). Pedigrees were produced for each cross by including dummy parents (one pair per cross) from which all F2s were descended. Genotype likelihoods were called from the VCF input with the *ParentCall2* module, including the *-halfSibs=1* flag. Further filtering was performed with the *Filtering2* module removing markers with a MAF < 0.1 (within families) and with missing data in >10% of individuals (within families). Markers were mapped to linkage groups (LGs) with *SeperateChromosomes2* modules based on a logarithm of the odds (LOD) score of 20, using all informative markers, and grandparental phase information. LGs with fewer than 20 markers were discarded, leaving 21 LGs. The two largest LGs were separated by further iterations of *SeparateChromosomes2* run over these specific LGs with an elevated LOD limit of 30. This produced 23 LGs in agreement with the assembled guppy genome. Unmapped markers were joined to the 23 LGs with the *JoinSingles2All* module, with an LOD limit of 5. The module was iterated until no further markers could be mapped. In total, 7,256 markers of 16,539 were mapped to LGs. The module *OrderMarkers2* was then run over each LG independently to order and place markers within LGs. An initial 10 iterations were performed, with order determined by maximum likelihood. For chromosome 12, male recombination was not permitted given previous evidence that males do not recombine over the sex chromosome (Charlesworth, Zhang, *et al.*, 2020). LOD

scores from these maps were used to further filter markers on the basis of support for multiple mappings within a LG (multiple LOD peaks) or if maximum LOD was within one standard deviation of the mean. These markers were blacklisted for the final *OrderMarkers2* run, in which the *evaluateOrder* flag was run over the earlier maximum-likelihood based map. Final maps were sex-averaged and trimmed according to graphical evaluation. Grandparental-phased genotypes were exported for QTL analysis.

***Heterochiasmy and linkage mapping***

There were some differences between male and female maps prior to averaging on some linkage groups, which is expected given female-biased heterochiasmy (Bergero et al. 2019), however these differences were minor and generally reflected differences of intercept rather than slope when comparing genetic maps to the physical genome. These sex-averaged maps therefore are accurate representations of the relative genetic map, but are limited in terms of inferring accurate estimates of recombination rate.

***QTL and GCTA-GREML statistical power analyses***

We assessed the power of our sampling design to detect QTLs of varying heritability (h2), in particular the sample sizes used for females (n=267) and males (n=370). Analysis was performed following the derivations of Wang & Xu (2019), using a matrix of the fully-informative QTL markers (n=1220). Effective sample size was calculated using the eigenvalues of a normalised QTL kinship matrix, under $\lambda=1$, using this to calculate the effective correlation coefficient among the samples (rho). We first calculated our power to detect QTL sizes of h2=0.01 to h2=0.1 (0.01 increments) using our empirical sample size and marker number. We also calculated the power of varying sample sizes required to detect QTL sizes of h2=0.01 to h2=0.1 (0.01 increments) under power ranging from 0.1 to 0.99 (0.1 increments), using rho and our marker number. For GCTA-GREML power, we used the online GCTA-GREML Power Calculator shiny app. We estimated statistical power associated with a range of h2 from 0.01 to 0.5, using sample sizes of n=370 (males) and n=267 (females), and an alpha threshold of 0.05. Variance of SNP-derived genetic relationships was calculated as the variance of genetic

relatedness estimates between individuals in the relevant genetic relatedness matrices (GRMs), excluding comparisons made within families.

## SUPPLEMENTARY RESULTS:

### ***Full analysis of candidate genes***

The QTL at chr19:66.954, associated with offspring weight, covered ~8.2 Mb of chromosome 19, and included 267 genes. Due to the large size of this region, and that alternative confidence intervals (Bayesian 5% probability intervals) suggested a single peak at chr19:66.954, we limited our curation of candidate genes to the immediate 0.5 Mb either side of the peak (chr19:18602889-19602889). This region included 40 genes. Several of these genes (*wfikkn2b*, *tob1b*, *sap30bp*, *h3-3b*, *unk*, *mrpl38*, *fdxr*, *narf*, *cybc1*) are expressed in all stages of embryonic development in zebrafish, or interact with growth signalling pathways, suggesting potential functional effects for offspring weight. The closest gene to the peak was *cdr2l*. Of the 40 genes in this region, 18 exhibited female-biased differential expression in gonads based on the guppy transcriptome (Sharma et al. 2014), and six exhibited male-biased expression (Table S11), suggesting potential reproductive function.

The chr22 QTL peak (for size at first brood) was on a previously unplaced scaffold 000111F_0:651336 and confidence intervals extended over the scaffold (00111F_0:528180-1199617) and a region at the distal end of chromosome 22 (chr22:23415429-24223839). The peak of the QTL did not overlap any genes, however the closest gene was F-box protein 33, *fbxo33*. In total, the region included 45 genes. There were no genes with GO terms or KEGG annotations indicative of clear roles in growth. According to the GeneCards database however, several genes had evidence of affecting size and growth phenotypes in mice knockout studies (*fbxo33*, *tonsl*, *cnr1*, *cga*, *htr1b* and *myo6*). Two of the genes (*cga* and *htr1b*) in this region are also associated with various hormonal pathways, including gonadotropin hormone signalling that may affect development. Other genes in this region are associated with myogenesis, including *myo6* and genes within the Akirin family (*akirin2*, *gabrr1*, *pm20d2*, *cnr1*, *syncripl*). In

addition, the gene *snx14* has been associated with growth QTLs in grass carp (Huang et al. 2020).

The interbrood period QTL on chr12 overlapped the YTH domain containing 1 gene, *ythdc1*, on a previously unplaced scaffold 000149F_0 (000149F_0: 131600). This scaffold was placed at the distal end of chromosome 12 near the sex-determining region, and corresponds with scaffold KK215301.1 in the older female genome (Künstner et al. 2016), which has similarly been placed at the distal end of chromosome 12 in other mapping studies (Charlesworth et al. 2020). The confidence interval around this QTL covered additional regions of scaffold 000149F_0 (000149F_0:47124-197258) and chromosome 12 (chr12:24525856-24705290). This region included 18 genes, but none were associated with clear GO or KEGG terms indicative of roles for female fertility. Four genes exhibited female-biased expression in gonads and seven were male-biased. The gene overlapping the QTL peak, *ythdc1*, however is a promising candidate due to its interactions with N6-methyladenosine ($m^6A$) (Xia et al. 2018; X. Wang et al. 2015). Disruption of $m^6A$ by mutation of another modifier *mettl3*, affected oocyte development and reduced the proportion of full-growth follicles in zebrafish (Xia et al. 2018). Further, *Ythdc1*-deficient mice have oocyte maturation blocked at the primary follicle stage and experience alternative splicing defects in oocytes (Kasowitz et al. 2018). Examination of transcripts matching *ythdc1* (largest = CUFF_24477_m.316355) in the guppy transcriptome (2014) revealed significant overexpression in ovaries compared with testes. Sequence analysis alongside other Poeciliids (Ensembl release 101: *P. formosa, P. latipinna, P. mexicana,* and *Xiphophorus maculeatus*) demonstrated significant purifying selection on this gene (dN/dS ≤ 0.197; Z-tests for purifying selection: $2.79 \leq Z \leq 7.40$; Table S12). These provide strong evidence for a functional reproductive role for this gene in guppies.

## REFERENCES

Baird, Nathan A., Paul D. Etter, Tressa S. Atwood, Mark C. Currey, Anthony L. Shiver, Zachary A. Lewis, Eric U. Selker, William A. Cresko, and Eric A. Johnson. 2008. “Rapid SNP Discovery

and Genetic Mapping Using Sequenced RAD Markers." *PloS One* 3 (10): e3376.

Bergero, Roberta, Jim Gardner, Beth Bader, Lengxob Yong, and Deborah Charlesworth. 2019. "Exaggerated Heterochiasmy in a Fish with Sex-Linked Male Coloration Polymorphisms." *Proceedings of the National Academy of Sciences of the United States of America* 116 (14): 6924–31.

Charlesworth, Deborah, Roberta Bergero, Chay Graham, Jim Gardner, and Lengxob Yong. 2020. "Locating the Sex Determining Region of Linkage Group 12 of Guppy (Poecilia Reticulata)."

Huang, Xiaoli, Yanxin Jiang, Wanting Zhang, Yingyin Cheng, Yaping Wang, Xiaocui Ma, You Duan, et al. 2020. "Construction of a High-Density Genetic Map and Mapping of Growth Related QTLs in the Grass Carp (Ctenopharyngodon Idellus)." *BMC Genomics* 21 (1): 313.

Kasowitz, Seth D., Jun Ma, Stephen J. Anderson, N. Adrian Leu, Yang Xu, Brian D. Gregory, Richard M. Schultz, and P. Jeremy Wang. 2018. "Nuclear m6A Reader YTHDC1 Regulates Alternative Polyadenylation and Splicing during Mouse Oocyte Development." *PLoS Genetics* 14 (5): e1007412.

Künstner, Axel, Margarete Hoffmann, Bonnie A. Fraser, Verena A. Kottler, Eshita Sharma, Detlef Weigel, and Christine Dreyer. 2016. "The Genome of the Trinidadian Guppy , Poecilia Reticulata , and Variation in the Guanapo Population." *PloS One*, 1–25.

Miller, Michael R., Joseph P. Dunham, Angel Amores, William A. Cresko, and Eric A. Johnson. 2007. "Rapid and Cost-Effective Polymorphism Identification and Genotyping Using Restriction Site Associated DNA (RAD) Markers." *Genome Research* 17 (2): 240–48.

Nicholls, James A., Michael C. Double, David M. Rowell, and Robert D. Magrath. 2000. "The Evolution of Cooperative and Pair Breeding in Thornbills Acanthiza (Pardalotidae)." *Journal of Avian Biology* 31 (2): 165–76.

Poland, Jesse A., Patrick J. Brown, Mark E. Sorrells, and Jean-Luc Jannink. 2012. "Development of High-Density Genetic Maps for Barley and Wheat Using a Novel Two-Enzyme Genotyping-by-Sequencing Approach." *PloS One* 7 (2): e32253.

Reznick, David. 1982. "The Impact of Predation on Life History Evolution in Trinidadian Guppies: Genetic Basis of Observed Life History Patterns." *Evolution; International Journal of Organic Evolution* 36 (6): 1236–50.

Richardson, D. S., F. L. Jury, K. Blaakmeer, J. Komdeur, and T. Burke. 2001. "Parentage Assignment and Extra-Group Paternity in a Cooperative Breeder: The Seychelles Warbler (Acrocephalus Sechellensis)." *Molecular Ecology* 10 (9): 2263–73.

Sharma, Eshita, Axel Künstner, Bonnie A. Fraser, Gideon Zipprich, Verena A. Kottler, Stefan R. Henz, Detlef Weigel, and Christine Dreyer. 2014. "Transcriptome Assemblies for Studying Sex-Biased Gene Expression in the Guppy, Poecilia Reticulata." *BMC Genomics* 15 (May): 400.

Wang, Meiyue, and Shizhong Xu. 2019. "Statistical Power in Genome-Wide Association Studies and Quantitative Trait Locus Mapping." *Heredity* 123 (3): 287–306.

Wang, Xiao, Boxuan Simen Zhao, Ian A. Roundtree, Zhike Lu, Dali Han, Honghui Ma, Xiaocheng Weng, Kai Chen, Hailing Shi, and Chuan He. 2015. "N(6)-Methyladenosine Modulates Messenger RNA Translation Efficiency." *Cell* 161 (6): 1388–99.

Xia, Hui, Chengrong Zhong, Xingxing Wu, Ji Chen, Binbin Tao, Xiaoqin Xia, Mijuan Shi, Zuoyan Zhu, Vance L. Trudeau, and Wei Hu. 2018. "Mettl3 Mutation Disrupts Gamete Maturation and Reduces Fertility in Zebrafish." *Genetics* 208 (2): 729–43.

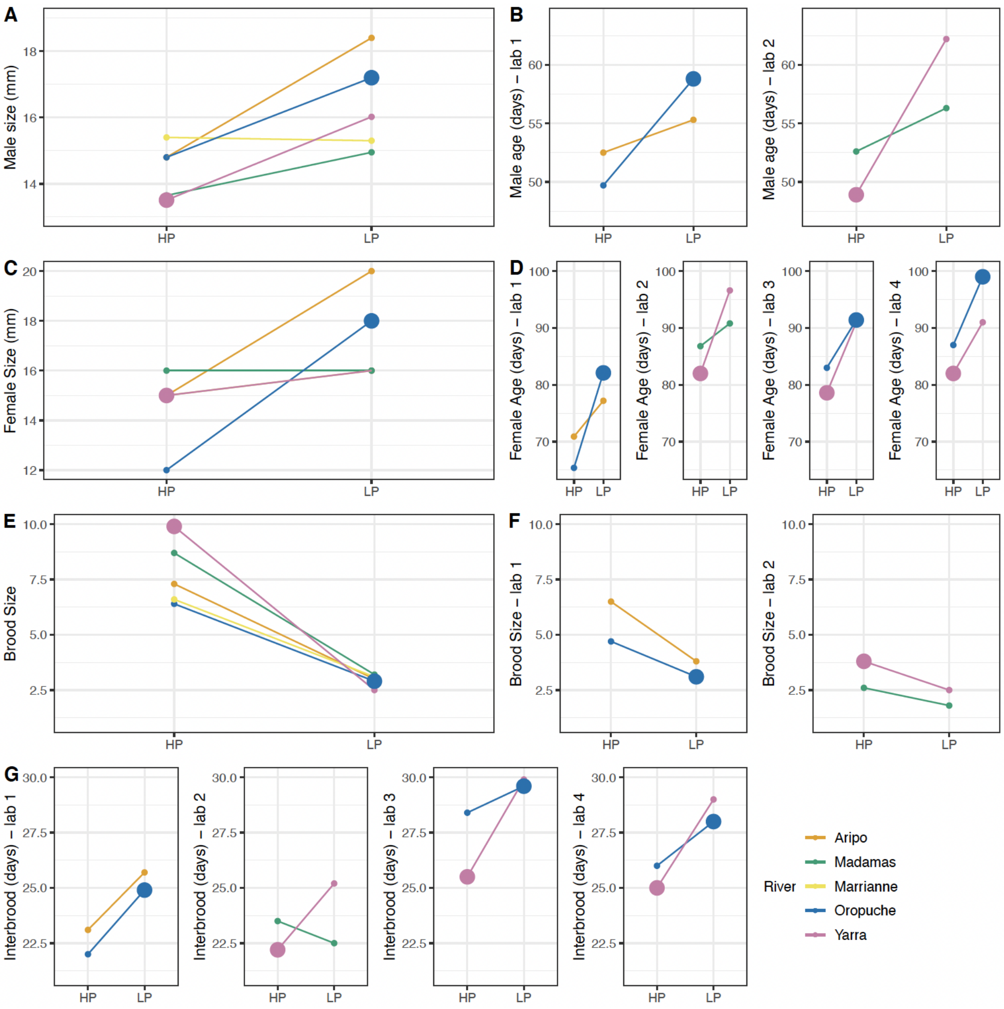


**Figure S1:** Summary of published life history traits for paired high predation (HP) and low predation (LP) populations within rivers. (**A**) size of mature males (**B**) male age at maturity (**C**) female size, as estimated by minimum size of gravid females (**D**) female age at maturity (**E**) brood size as estimates as number of offspring for 30 mg females (**F**) brood size of first brood (**G**) interbrood interval between first and second broods. Data in (**A**), (**C**), and (**E**) are from wild caught individuals and are found in Reznick and Endler 1982 Evolution, and Reznick et al. 1996 Am Nat. Data from (**B**), (**D**), (**F**), and (**G**) are from lab reared studies in Reznick 1982 Evolution (lab1), Reznick and Bryga 1996 (lab2), Reznick et al. 2005 PLoS Biol (lab3) and Reznick et al. 2001 Exp Geren. (lab4). Lab data is plotted separately due to the large effect of rearing environment on phenotypes. Data for the high food rations were plotted because it more closely reflected data in the current paper. Focal populations for our QTL cross are indicated with larger circles.

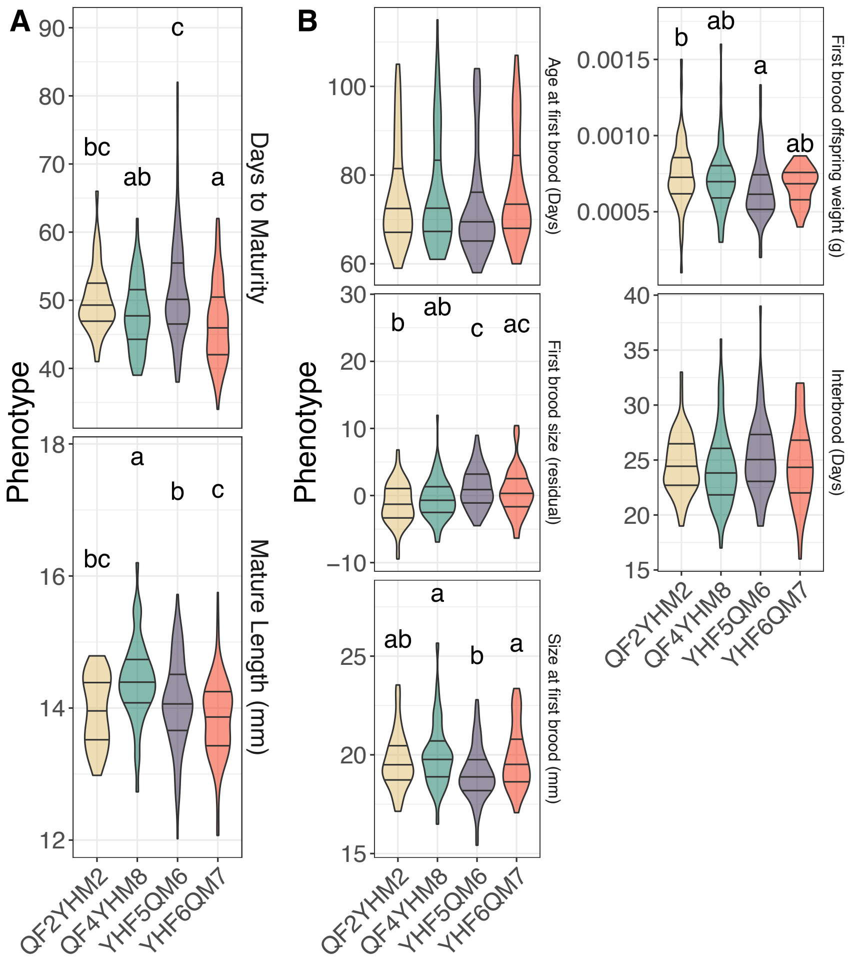


**Figure S2:** Phenotype distributions for male (**A**) and female (**B**) life history phenotypes grouped between families. Where phenotypes differed significantly between families, significance groups are highlighted above violins. Each violin shows the median and quartiles.

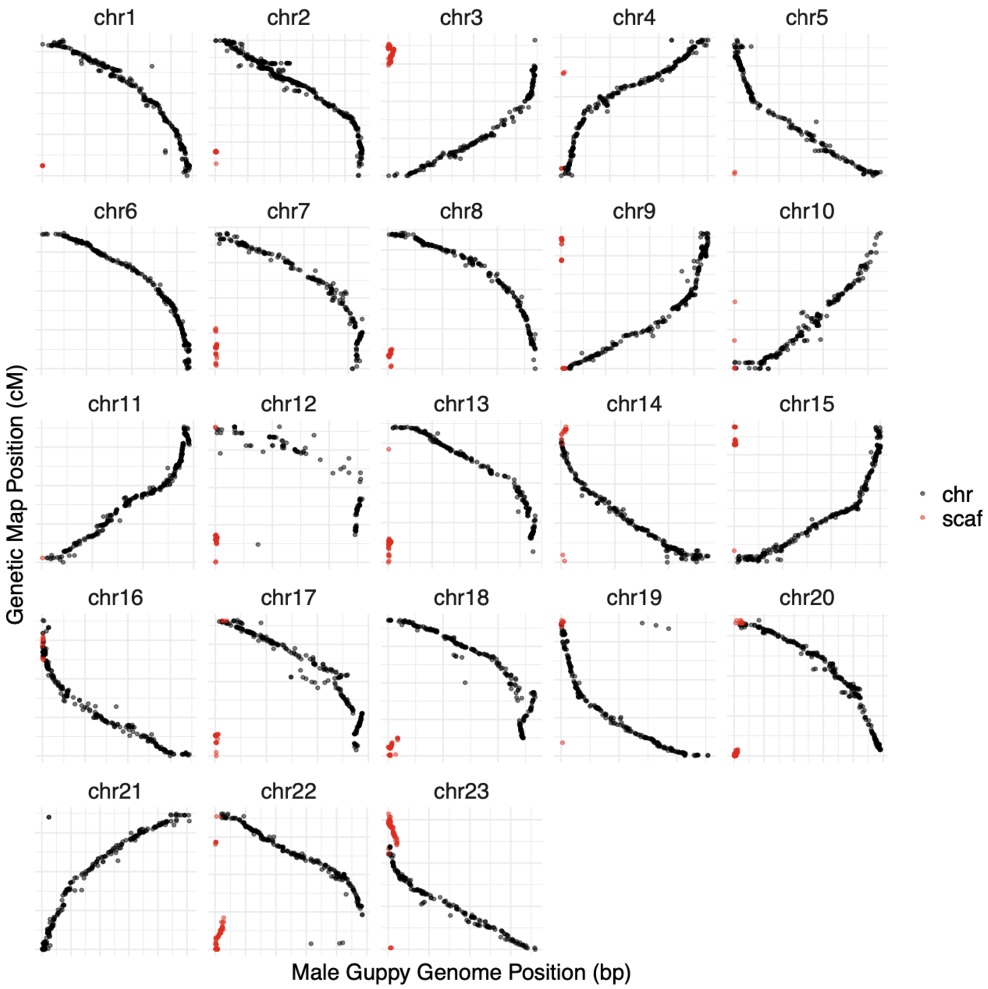


**Figure S3**: Comparison of genetic map positions against male guppy genome. Each facet highlights one of the 23 chromosomes in the genome. Black points represent markers on scaffolds previously mapped to chromosomes, markers in red highlight additional chromosomal regions made up of markers from unplaced scaffolds (Table S2). Positions on the x-axis represent genome positions in bp, always starting at 0. Y coordinates reflect genetic map position, which in some cases is reversed relative to the published genome. Genetic map positions were reversed to match published genome order for final estimates of QTL loci and whole-genome visualisations of QTL LOD distributions.

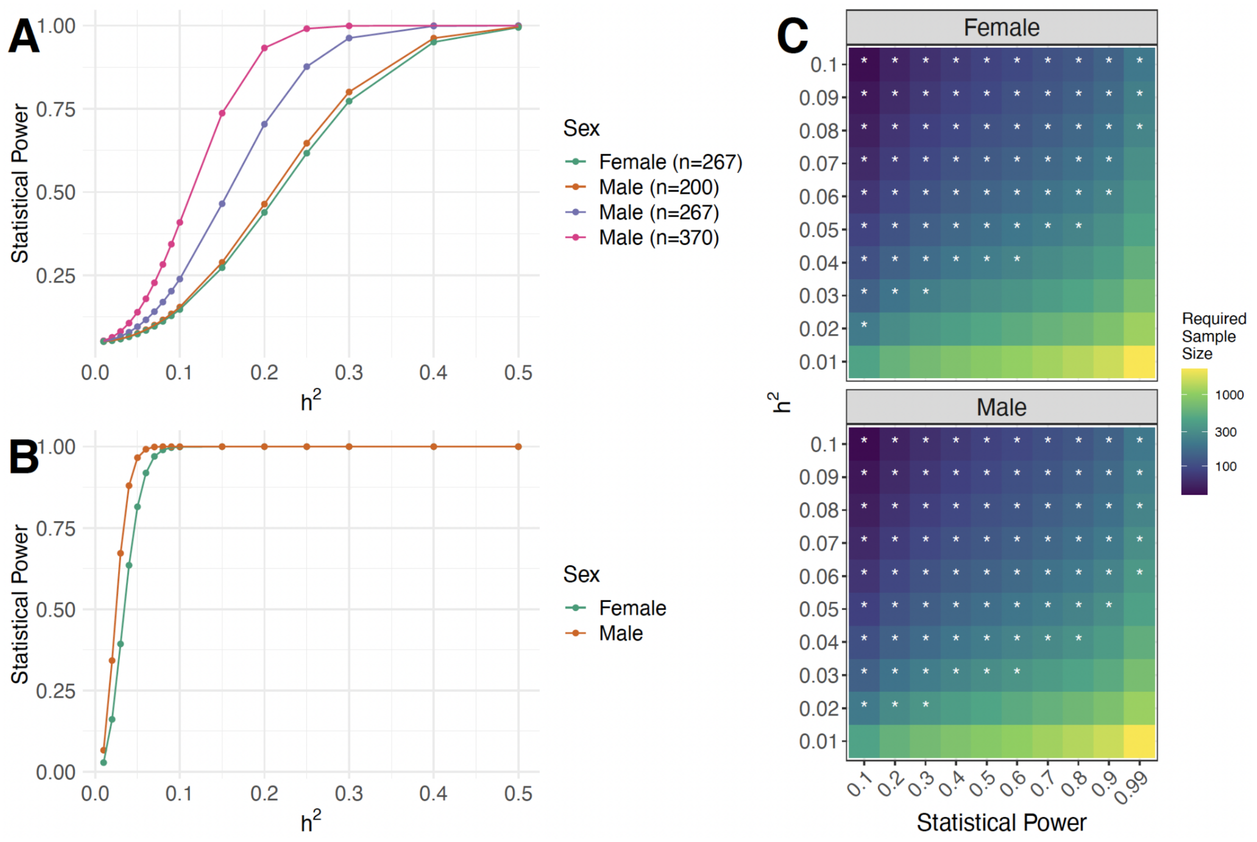


**Figure S4:** Results of power analyses for GCTA-GREML (A) and QTL scans (B and C). Panels A and B show the statistical power associated with detected genome-wide signal of heritability (A) or a QTL of varying effect size (B) based on our male and female marker sets. Panel C shows the required sample sizes to detect QTL of varying effect sizes at varying levels of statistical power in our fully-informative (n=1220) marker set for males and females. White asterisks denote cells for which the required sample size is less than or equal to our actual sample sizes (females = 267, males = 370).

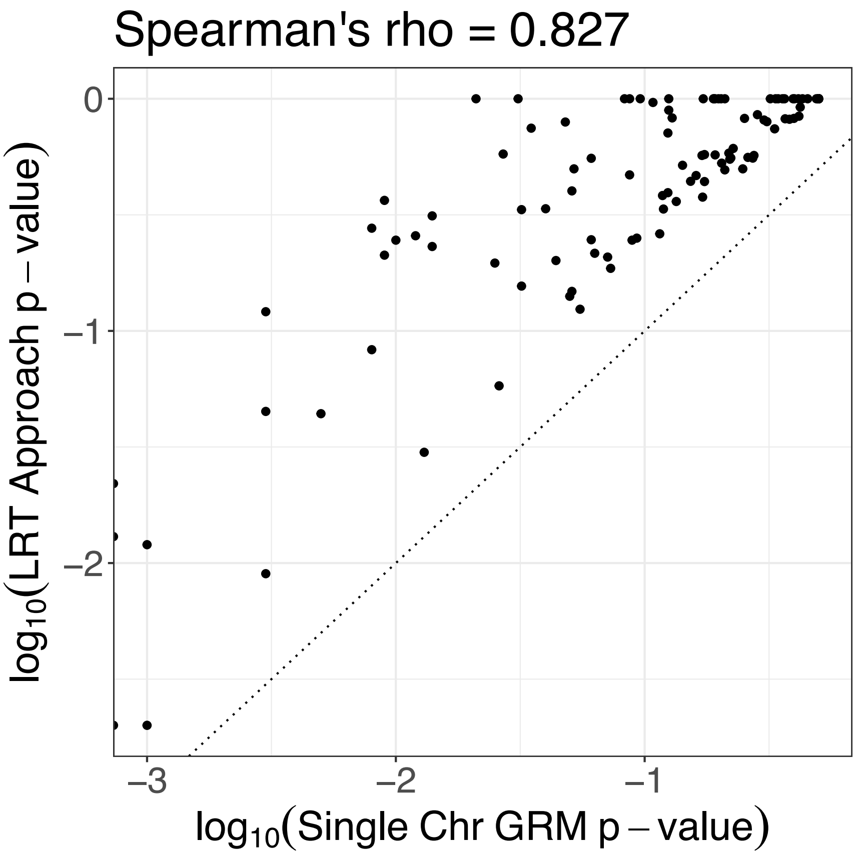


**Figure S5**: Linear relationship between log10-transformed p-values derived from either single chromosome GRM and LRT-approach estimates of $h^2c$. The y=x relationship is shown as a dotted line. Downward-biasing of single chromosome GRM p-values is evidenced as a shift off the y=x.

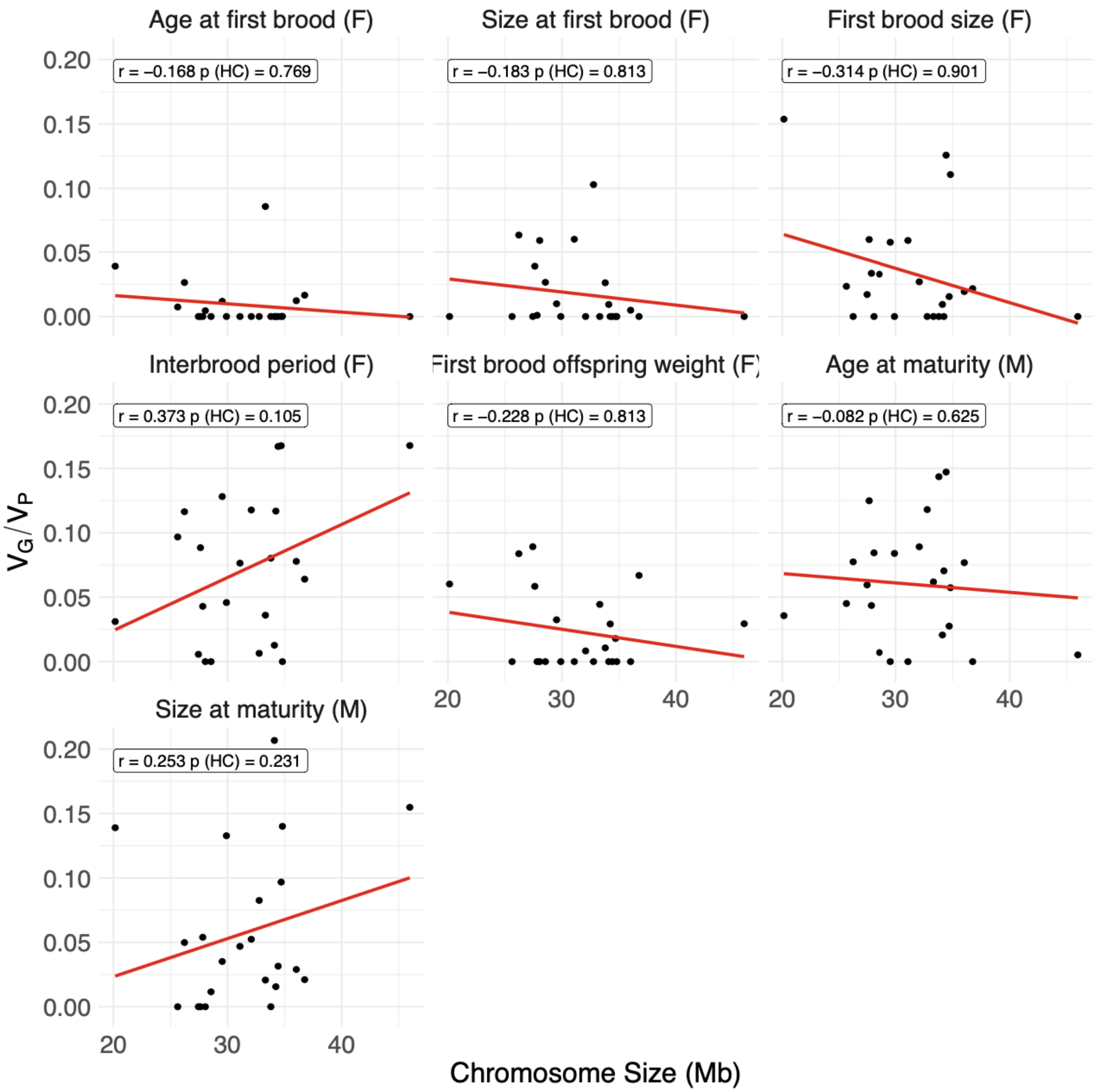


**Figure S6**: Correlations between single chromosome GRM $h^2c$ estimates and chromosome size. Chromosome size was estimated at the cumulative size of chromosomes and any scaffolds that could be merged to chromosomes according to the linkage map (Figure S3). HC-correction was performed following Kemppainen and Husby (2018), and HC-corrected p-values are shown alongside Pearson's correlation coefficients within each facet.

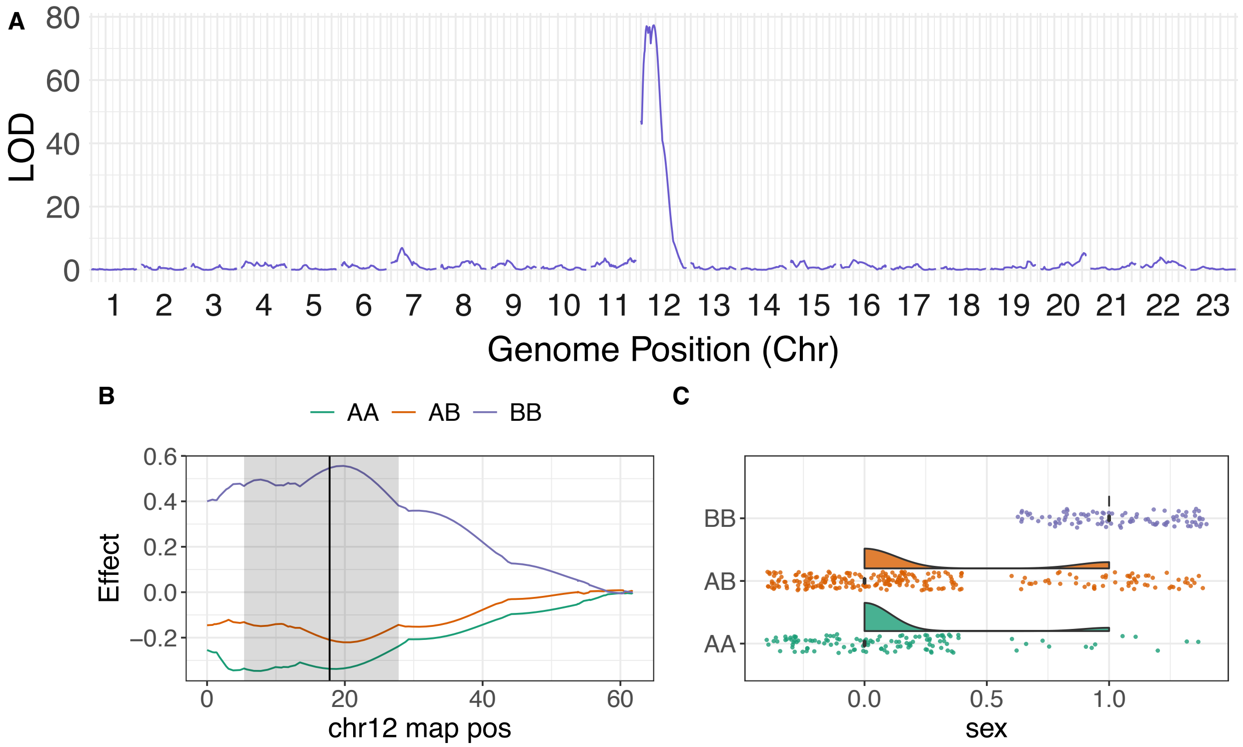


**Figure S7**: Single-locus QTL scan for binary sex classification across all males and females. Models were performed on a binary trait, where male = 0 and female = 1. For this model, genotypes were labelled such that “A” genotypes were inherited from the grandfather, and “B” genotypes were inherited from the grandmother, regardless of cross direction. Panel **B** shows genotype effects across LG12, with the peak denoted as a black line with grey shaded regions showing confidence intervals (drop LOD = 1.5). Panel **C** shows distributions of sex at the peak between the three genotypes, and highlights the expected distribution of a Y-linked marker, such that males cannot inherit both B alleles from the grandmother.

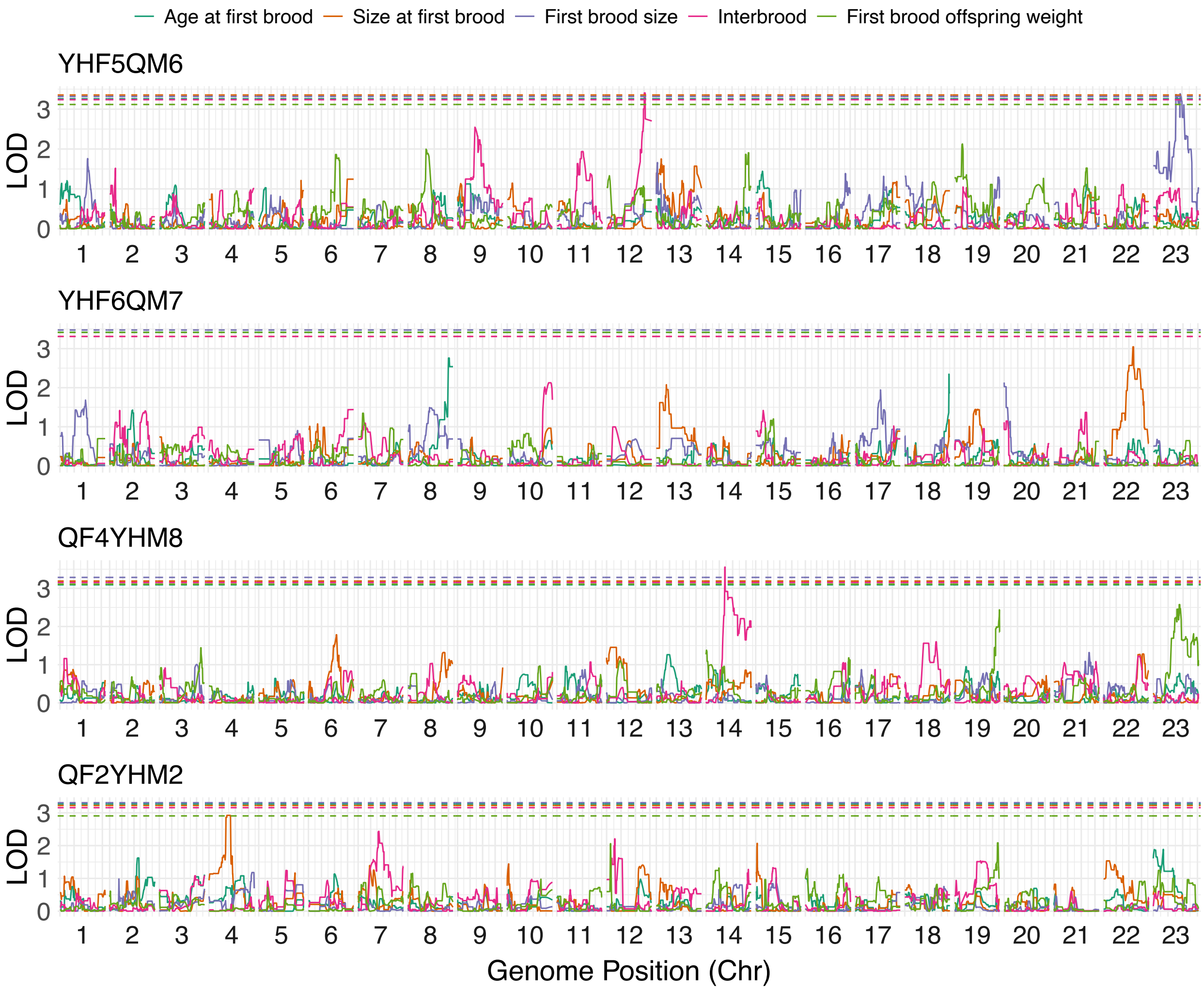


**Figure S8**: Within-family single-locus QTL scans for all five female life history traits. Each row highlights LOD scores across the genome for each of the four crosses. Where significant QTL were detected, 5% permuted (N = 1000) significance thresholds are shown as dashed lines. This analysis highlighted three within-family QTL: two in YHF5QM6, and one in QF4YHM8.

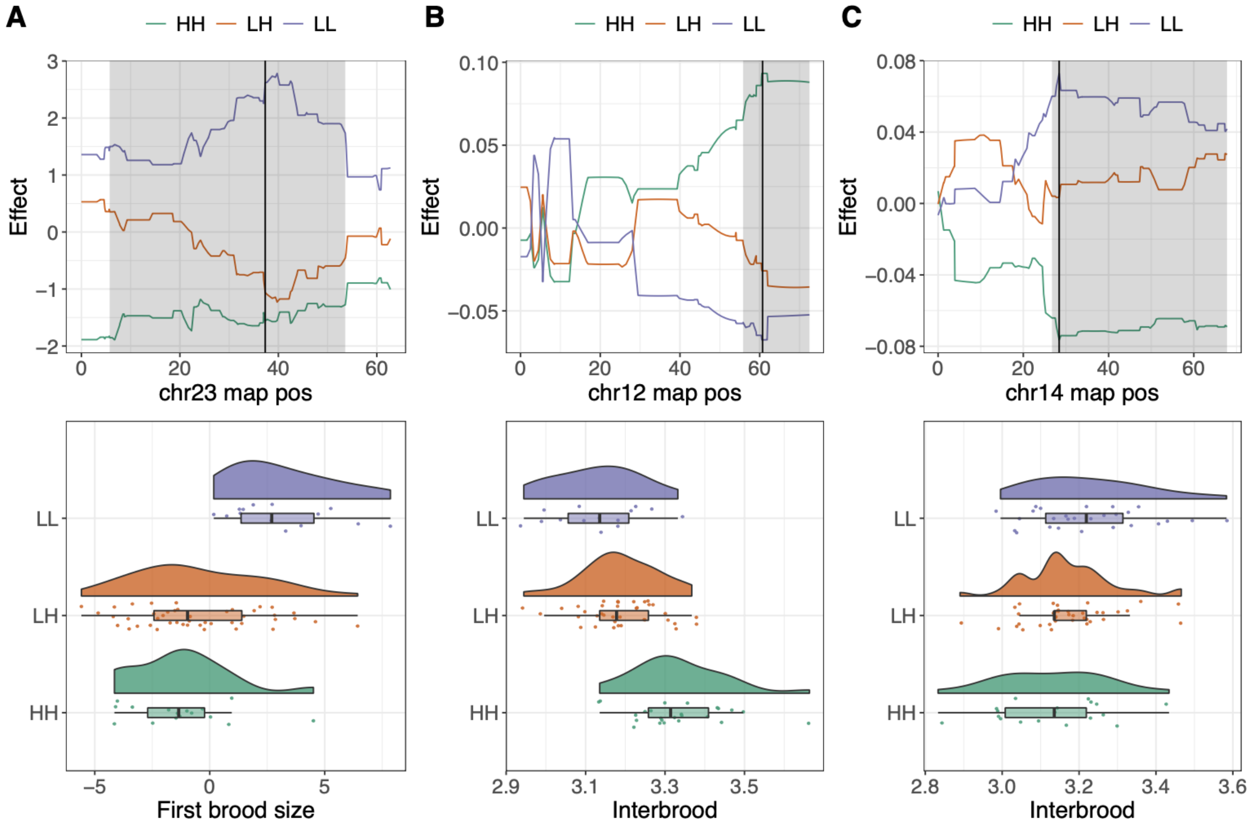


**Figure S9**: QTL effect plots for female within-family QTL detected in families YHF5QM6 (**A**-**B**) and QF4YHM8 (**C**). Each column shows QTL effects across the focal linkage groups (first row), and distributions of phenotypes across genotypes at the peak (second row). For focal chromosomes, the QTL peak is shown as a black line, with confidence intervals (LOD drop = 1.5) highlighted by grey shaded areas. QTL were associated with first brood size (**A**) and female interbrood period (**B**-**C**).

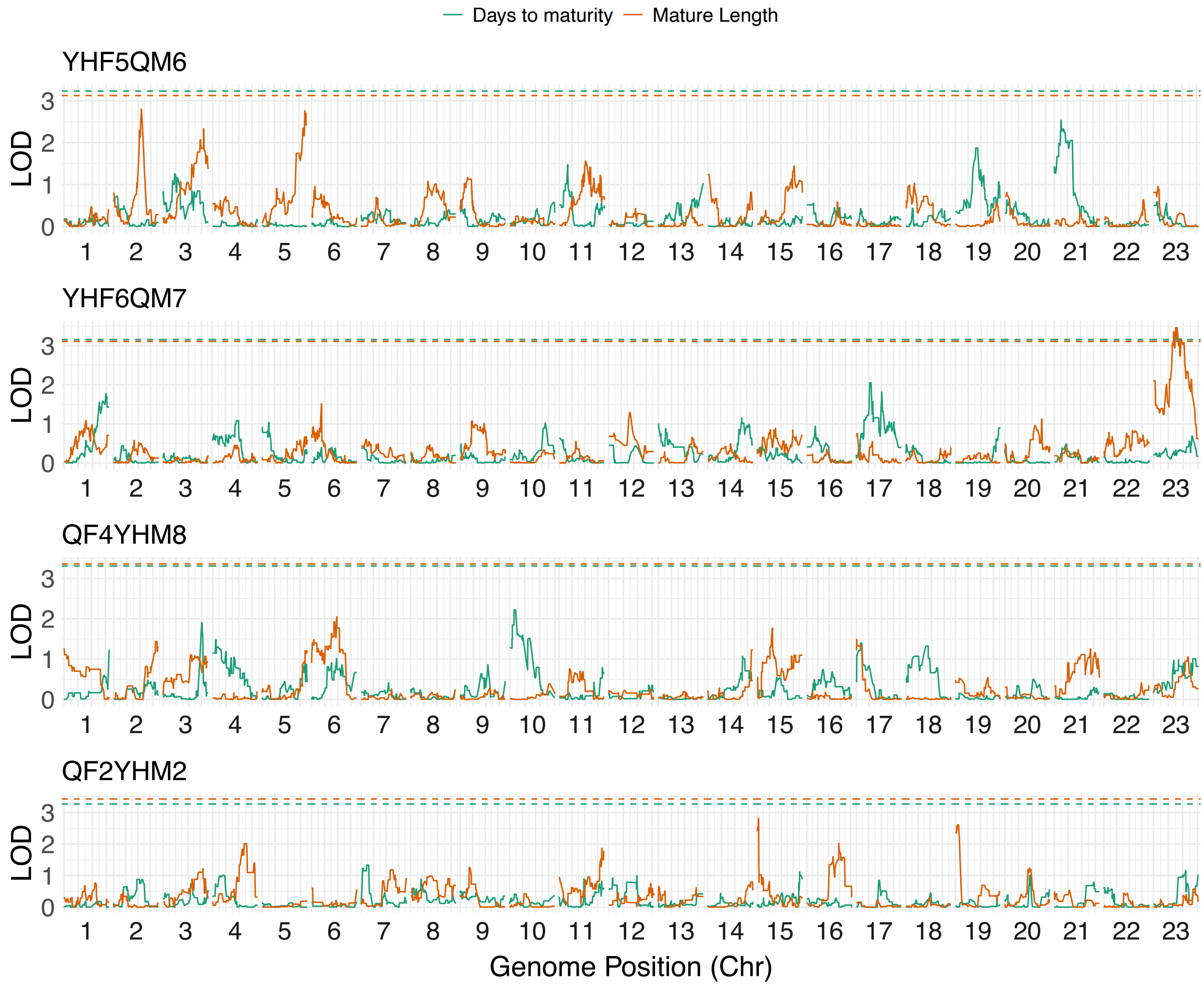


**Figure S10**: Within-family single-locus QTL scans for the two male life history traits. Each row highlights LOD scores across the genome for each of the four crosses. Where significant QTL were detected, 5% permuted (N = 1000) significance thresholds are shown as dashed lines. This analysis highlighted one within-family QTL in YHF6QM7.

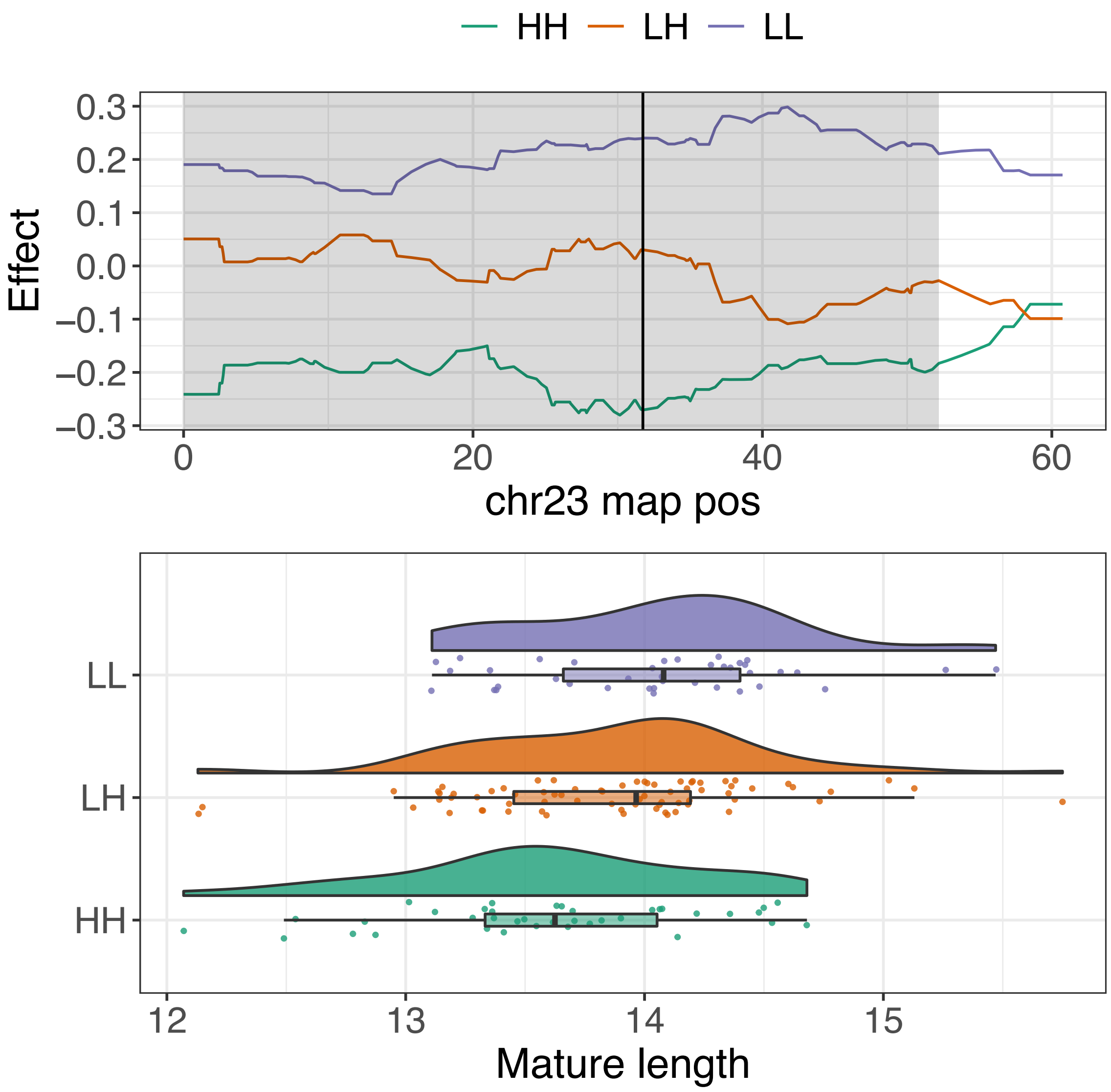


**Figure S11**: QTL effect plot for the male within-family QTL detected in family YHF6QM7**.** The first row shows QTL effects across the focal linkage groups, and the second row shows the distributions of phenotypes across genotypes at the peak. For the focal chromosome, the QTL peak is shown as a black line, with confidence intervals (LOD drop = 1.5) highlighted by grey shaded areas. This QTL was associated with male length at maturity.

**Table S6:** Principal component analysis between female phenotypes, highlighting positive covariance among age and size at first brood and first brood size.

| Phenotype | PC1: 37.6% | PC2: 27% | PC3: 17.7% | PC4: 13.4% | PC5: 4.3% |
|---|---|---|---|---|---|
| Age at first brood | 0.684 | -0.023 | -0.052 | -0.086 | -0.722 |
| Size at first brood | 0.58 | 0.278 | -0.399 | -0.26 | 0.6 |
| First brood size (residual) | 0.251 | -0.618 | -0.222 | 0.684 | 0.192 |
| Interbrood period | 0.208 | -0.587 | 0.565 | -0.489 | 0.234 |
| Offspring weight | 0.299 | 0.443 | 0.686 | 0.467 | 0.164 |

**Table S12**: dN/dS values associated with the *ythdc1* gene across the guppy and its close relatives.

| | *Poecilia reticulata* | *P formosa* | *P latipinna* | *P mexicana* | *Xiphophorus maculatus* |
|---|---|---|---|---|---|
| *P reticulata* | 0 | 0.167 | 0.19 | 0.194 | 0.197 |
| *P formosa* | 0.167 | 0 | 0.11 | 0.114 | 0.12 |
| *P latipinna* | 0.19 | 0.11 | 0 | 0.111 | 0.148 |
| *P mexicana* | 0.194 | 0.114 | 0.111 | 0 | 0.111 |
| *X maculatus* | 0.197 | 0.12 | 0.148 | 0.111 | 0 |
